# Dengue Virus Infection Perturbs Lipid Homeostasis in Infected Mosquito Cells

**DOI:** 10.1371/journal.ppat.1002584

**Published:** 2012-03-22

**Authors:** Rushika Perera, Catherine Riley, Giorgis Isaac, Amber S. Hopf-Jannasch, Ronald J. Moore, Karl W. Weitz, Ljiljana Pasa-Tolic, Thomas O. Metz, Jiri Adamec, Richard J. Kuhn

**Affiliations:** 1 Markey Center for Structural Biology, Department of Biological Sciences, Purdue University, West Lafayette, Indiana, United States of America; 2 Biological Sciences Division, Pacific Northwest National Laboratory, Richland, Washington, United States of America; 3 Bindley Bioscience Center, Purdue University, West Lafayette, Indiana, United States of America; 4 Environmental Molecular Sciences Laboratory, Pacific Northwest National Laboratory, Richland, Washington, United States of America; University of North Carolina at Chapel Hill, United States of America

## Abstract

Dengue virus causes ∼50–100 million infections per year and thus is considered one of the most aggressive arthropod-borne human pathogen worldwide. During its replication, dengue virus induces dramatic alterations in the intracellular membranes of infected cells. This phenomenon is observed both in human and vector-derived cells. Using high-resolution mass spectrometry of mosquito cells, we show that this membrane remodeling is directly linked to a unique lipid repertoire induced by dengue virus infection. Specifically, 15% of the metabolites detected were significantly different between DENV infected and uninfected cells while 85% of the metabolites detected were significantly different in isolated replication complex membranes. Furthermore, we demonstrate that intracellular lipid redistribution induced by the inhibition of fatty acid synthase, the rate-limiting enzyme in lipid biosynthesis, is sufficient for cell survival but is inhibitory to dengue virus replication. Lipids that have the capacity to destabilize and change the curvature of membranes as well as lipids that change the permeability of membranes are enriched in dengue virus infected cells. Several sphingolipids and other bioactive signaling molecules that are involved in controlling membrane fusion, fission, and trafficking as well as molecules that influence cytoskeletal reorganization are also up regulated during dengue infection. These observations shed light on the emerging role of lipids in shaping the membrane and protein environments during viral infections and suggest membrane-organizing principles that may influence virus-induced intracellular membrane architecture.

## Author Summary

Dengue virus is one of the most aggressive human pathogens worldwide. It causes 50–100 million infections per year but there is no vaccine or antiviral that is currently effective against the disease. The virus is spread by *Aedes aegyptii* and *Aedes albopictus* mosquitoes and viral replication within the mosquito vector is required for transmission to a new human host. During this replication cycle, the virus causes significant changes to the membrane organization of infected cells. These virus-induced membrane alterations help to assemble arrays of viral replication factories and aid the virus to evade host antiviral defense mechanisms. Previously, much effort has been placed in trying to identify viral and cellular protein effectors that aid virus replication. In this study we have explored the role of lipids in the formation of these extensive membrane platforms in mosquito cells. Using high-resolution mass spectrometry we have profiled the lipid composition of dengue virus infected mosquito cells and compared it to uninfected cells. Through this we have identified several lipid classes that are differentially regulated during dengue virus replication. Using inhibitors of lipid biosynthesis we have also identified a lipid repertoire that is inhibitory to viral replication. Knowledge of how dengue virus utilizes cellular lipids and downstream signaling pathways to facilitate its replication will provide novel targets that could be utilized for developing effective antivirals. This study is also a forerunner for future comparative analyses of the human host and vector membrane environments required for viral replication.

## Introduction

In the past 20 years, it has become increasingly evident that lipids are important bioactive molecules that mediate signalling cascades and regulatory events in the cell. The ability to synthesize lipids predisposes an organism to function as a host to parasites that have lost or lack this trait [1]. Viruses as obligate parasites rely exclusively on the host to fulfill their membrane and lipid requirements. This is especially true for enveloped viruses since they utilize host-derived lipid membranes to facilitate release from infected cells by budding as well as to enter cells through membrane fusion. Lipids also form an integral structural component of the virus particle.

For most viruses that replicate in the cytoplasm of infected cells, lipids are essential for the replication of viral genomes. Both enveloped and non-enveloped viruses induce extensive ultrastructural changes in infected cells. Host-derived membranes are rearranged to provide extensive platforms that help to assemble arrays of replication factories [2]–[6]. Some of these factories are housed in specialized membrane compartments that assist in evading host antiviral defense mechanisms [2]–[4], [7]. These compartments also function to increase the local concentration of molecules necessary for efficient viral RNA replication and particle assembly. Recent advances in electron tomography techniques have been instrumental in providing a three-dimensional perspective of these virus-induced membranes [2]–[4], [7]. However, the metabolic cost to the host or vector and the contribution of lipid biosynthesis and trafficking to the formation of these replication factories is yet in its early stages of investigation [8]–[12].

In this study, we have focused on the importance of lipid biosynthesis on dengue virus (DENV) replication. DENV is one of the most aggressive re-emerging pathogens worldwide [13]. Over two and a half billion people in more than 100 endemic countries are at risk for contracting dengue fever. Currently 50–100 million cases of dengue fever are estimated annually [14]. Since DENV replicates within the mosquito vector as well as the human host, the spread of the virus can be greatly reduced by controlling the vector. Much effort has been placed in understanding the dynamics of virus transmission and replication in the mosquito vector, including identification of host proteins in the midgut and salivary glands that are regulated by DENV infection [15]–[17]. Less is known about the global impact of DENV on host metabolic pathways.

Previous electron microscopy studies on DENV infected mosquito cells have shown that virus-induced membrane structures similar to those observed in human cells are prevalent [18]–[19]. This extensive requirement in both host and vector, for intracellular membranes that support viral RNA replication and assembly suggest that quantifiable changes may exist in the lipid repertoire of the infected cell to assist in the formation of these membranes. Identifying these lipid changes that occur during infection is a necessary first step to discovering how DENV and its constituent proteins modify the lipid metabolism of cells. The reason(s) for such modifications has yet to be described, but can be pursued with a knowledge of which lipid changes occur. Furthermore, novel therapeutics that modify or inhibit these lipid changes and lipid-protein interactions could conceivably result in inhibition of virus replication.

To investigate these possibilities, we used high-resolution mass spectrometry methods to profile the lipidome of DENV infected mosquito cells. We have identified several lipid classes that are regulated by DENV infection. Many of these lipids have characteristic roles in influencing membrane architecture as well as functioning in cellular signal transduction pathways. Specifically, we have identified differences induced in the lipid profile upon virus binding and entry alone compared to those induced by viral RNA replication, assembly and egress. We have also profiled the lipidome of cells treated with an inhibitor of de novo phospholipid biosynthesis. Through this we have identified a lipid environment that supports cell survival but is yet inhibitory to DENV replication.

## Results

**Figure 1:**
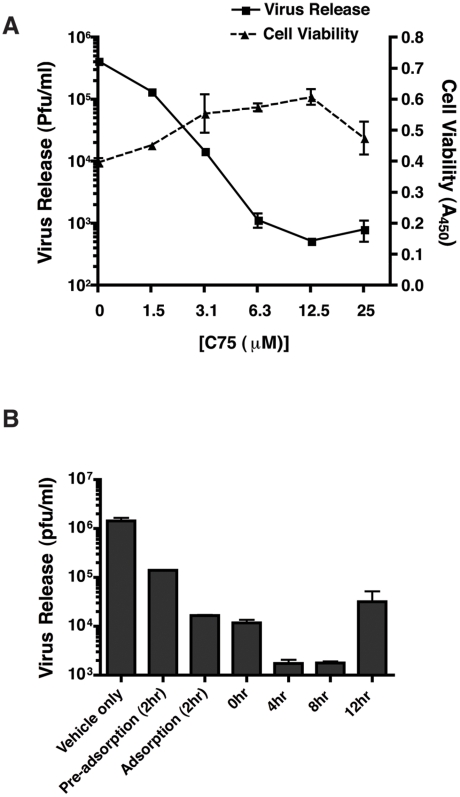
C75, a fatty acid synthase inhibitor disrupts DENV replication in mosquito cells. **A.** C6/36 mosquito cells were infected with DENV at an MOI of 3. Following adsorption of the virus, the indicated concentrations of C75 were added to the cells in the overlay media. A vehicle control of ethanol only was also included. Virus release was assayed by plaque assay 24 hr post-infection. Cell viability was assayed using the Quick Cell Proliferation Kit (Promega) at 24 hr post-infection. **B.** A time course of addition of C75 in C6/36 cells infected with DENV at an MOI of 3. Cells were either pre-treated (2 hr), or treated with 6.3 uM C75 during adsorption, post-adsorption (0 hr) or at 4, 8 and 12 hr post-infection. The results represent three independent experiments. The error bars represent standard deviation of the mean.

We had previously shown that fatty acid biosynthesis was a key target of DENV in human cells and that the rate limiting enzyme, fatty acid synthase (FAS), was both required and re-localized to sites of viral RNA replication during DENV infection [20]. In this study, using an inhibitor of FAS, C75, we determined that this requirement for fatty acid biosynthesis was conserved between the host and its vector during DENV infection. In the presence of C75 DENV replication was significantly reduced in C6/36 mosquito cells indicating that fatty acid biosynthesis is important for virus viability (Figure 1A). Furthermore, a time course of addition of C75 (Figure 1B) indicated that while pre-treatment or treatment of cells with C75 during viral adsorption reduced virus replication by ∼10–100 fold, the most significant effect occurred upon addition of the drug at 4 and 8 hr post-infection (∼1000 fold). This suggested that a post-entry step was affected by the inhibition of FAS. A comparison of virus released into the supernatant to intracellular virus indicated that an accumulation of intracellular virus was not occurring in the C75 treated cells (data not shown). Thus, the block in replication was not at the level of virus assembly or release.

### Analysis of the whole cell lipidome

Based on the observation that DENV induces significant rearrangements in the membrane architecture of infected cells, together with its distinct susceptibility to inhibitors of FAS, it was our hypothesis that lipid biosynthesis was important to DENV replication. Therefore, to investigate whether the intracellular lipid composition was altered during DENV infection, we carried out LC-MS-based analyses of the global lipidome of mosquito cells infected with DENV. To differentiate between changes to the lipid profile that occurred upon exposure to infectious virus versus that induced by virus entry alone, we included UV-inactivated DENV (UV-DENV) in the studies. This inactivated virus is only capable of binding, entry and initial rounds of viral RNA translation but does not have the ability to replicate its genome.

**Figure 2:**
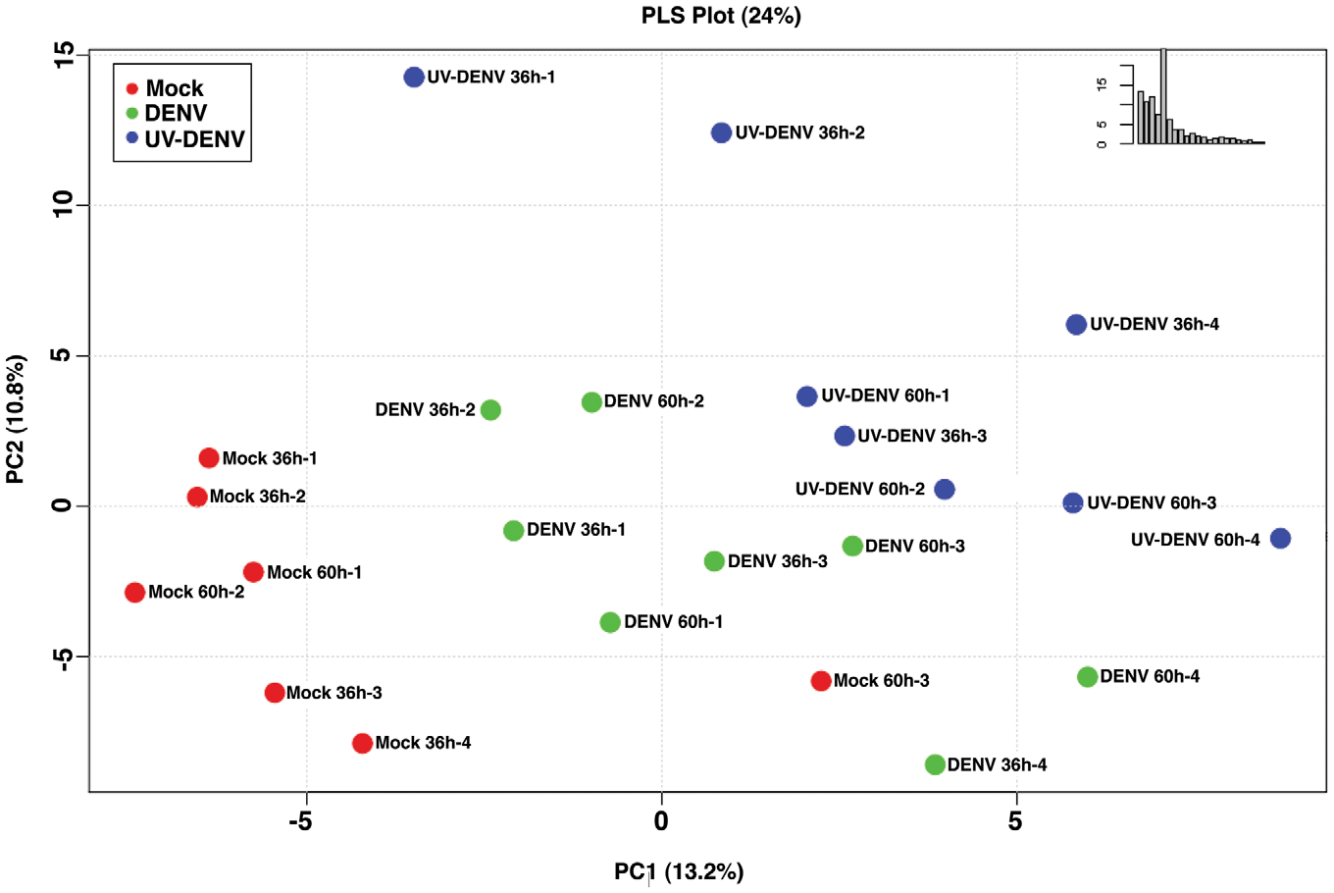
A plot of the principle components analysis scores shows segregation of the global lipid profile between uninfected and DENV-infected mosquito cells. The abundance of lipids in C6/36 cells infected with either DENV (MOI 20) or UV-DENV was measured at 36 and 60 hr post-infection and compared to uninfected controls (mock). Each experiment included 4 independent replicates. A total of 7217 features were compared by principle component analysis (PCA). The plot shows differences that are specific to infectious virus (DENV), a non-replicating virus (DENV-UV) and the mock control.

**Figure 3:**
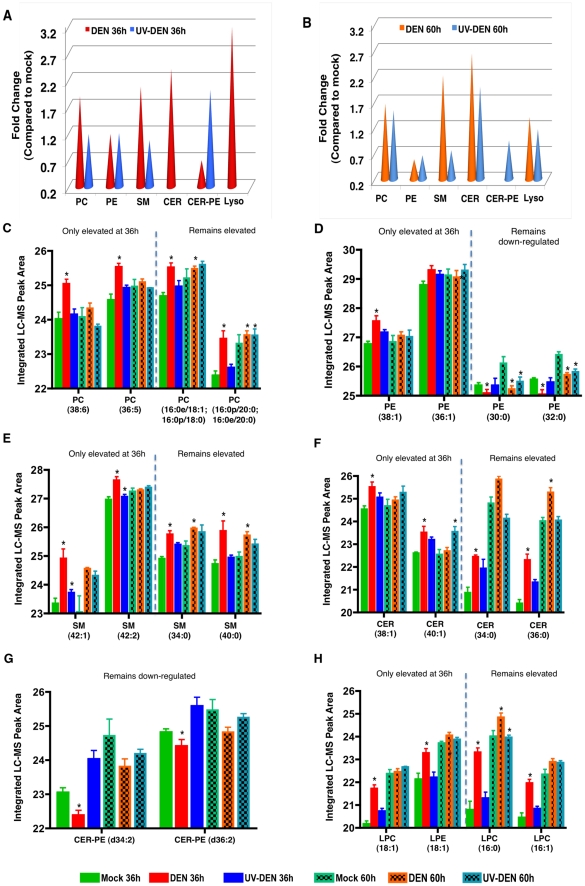
Whole cell lipidomics reveal an altered lipid composition in DENV infected cells. Panel A and B represent an average expression (fold change) of the total number of individual lipids significantly expressed (p<0.05) per lipid class at 36 and 60 hr post-infection, respectively. The fold changes represent DENV-infected cells or UV-DENV exposed cells compared to the mock control. A lack of cones indicates that the expression level of those specific lipids were not significant (p<0.05). Panels C–H are representative lipid molecular species from specific lipid classes significantly regulated at the two different time points. The data are plotted as the integrated LC-MS peak abundance, in log 2 scale with standard deviation. PC, phosphatidylcholine; PE, phosphatidylethanolamine; SM, sphingomyelin; CER, ceramide; CER-PE, ceramide phosphoethanolamine; Lyso, lysophospholipids. See supplementary table S1 for a complete list of lipid features detected in this study. Four replicates were included in the lipidomic analyses. The error bars represent standard deviation of the mean. The blue dashed line separates species that remain elevated at both time points (36 and 60 hr) from species that are only elevated at the 36 hr time point. Infections were carried out using an MOI of 20 in C6/36 cells. Significantly expressed lipids species are shown denoted with an asterisk (*).

Figure 2 shows a principle component analysis (PCA) plot of the overall lipid abundance in C6/36 mosquito cells infected with either DENV or UV-DENV at 36 and 60 hr post-infection. The 36 hr time point was chosen to represent a peak in viral replication while the 60 hr time point represents late stages of replication as well as increased cellular stress. Our previous analysis of earlier time points (ie. 24 hr post-infection) indicated that concurrent with increasing RNA synthesis activity and virus release, there were substantial changes in the lipidome of infected cells. Some of these changes were greater (higher fold changes) than those observed at the 36 hr time point. However, the overall intensities of the expressed lipids was lower contributing to a low signal to noise ratio. Furthermore, the number of species expressed at significant levels (p<0.05) were limited. Therefore, we chose to pursue the later time points. Under conditions that ensured that all cells were infected (multiplicity of infection of 20), optimal viral RNA synthesis occurred between 24–36 hr post infection (data not shown). A total of 7217 features observed in the LC-MS analyses were included in the PCA analyses. The plot shows specific segregation of lipid profiles between DENV and UV-DENV exposed cells compared to uninfected cells (mock). A temporal regulation of the lipid profile was also observed. However, this is more discernible upon analysis of individual lipid species (Figure 3) rather than in the PCA analysis. Overall, in this whole cell analysis 15% of the metabolites identified were significantly (Anova p<0.05) different between virus infected cells and the mock control.

Based on the total number of lipids that were significantly regulated between groups (uninfected and infected, Anova p<0.05) and subsequently structurally identified (Table S1 and Methods) the overall abundance of lipid classes is as follows: phosphotidylcholine (PC), 39.2%; phosphatidylethanolamine (PE), 31.2%; phosphatidylserine (PS), 0.8%; sphingomyelins (SM), 18.5%; ceramide (CER), 5.3%; lysophospholipids, 0.8% and ceramide phosphoethanolamine (CER-PE), 4%. This distribution is similar to the membrane lipid composition of eukaryotic cells where the most abundant phospholipid is PC [21]. It is also consistent with the membrane lipid composition of the *Diptera* species where PE is a predominant PL [22]. CER-PE, which is preferentially expressed in insect cells, is also observed here [23]–[24]. Several of these lipids are differentially regulated upon exposure of cells to DENV or UV-DENV (Figure 3). It was noticeable however, that several negatively charged lipid classes such as phosphatidylglycerol (PG), phosphatidylinositol (PI), and Cardiolipin, were absent from this list. These lipids have previously been reported to account for 6–13% of the lipidome of mosquito cells [25], [26]. However, in this whole cell analysis, these lipids were not significantly regulated during virus infection.

A comparative analysis of the overall abundance of lipid species (relative to the mock control) in DENV and UV-DENV exposed cells is shown in Figure 3A and B. In all of the lipid classes with significantly regulated lipids (Anova p<0.05), DENV-infected cells have a unique expression pattern (expressed lipid molecular species) compared to UV-DENV infected cells. For a complete list of the differentially expressed lipids see Table S1.

#### Phospholipids (PL)

In this whole cell analysis, the primary PLs that were significantly regulated (Anova p<0.05) (and subsequently structurally identified) were mostly neutral or zwitterionic. PS was the only acidic lipid significantly regulated. At 36 hr post-infection, there was an ∼2 fold up-regulation of PC species in DENV-infected cells compared to UV-DENV and mock controls (Figure 3A). At the later time point (60 hr post-infection), the relative levels of PC remained elevated in DENV-infected cells, however the differential expression between DENV and UV-DENV-exposed cells was not as evident (Figure 3B). Selected PC species were up regulated only at the 36 hr time point while others were up regulated at both time points (Figure 3C). Interestingly, a majority (∼80%) of the PC species that were up regulated had unsaturated fatty acyl chains.

Analysis of the overall fold change in PE at the 36 hr time point (Figure 3A) does not show a difference between the virus-exposed (DENV and UV-DENV) cells and the mock. This is due to an equal number of individual lipid molecular species being up regulated as were down regulated (Figure 3D). At the 60 hr time point the relative levels of PE in virus exposed cells were lower than mock levels (Figure 3B and D). There was also limited overlap between the specific PE molecular species regulated between DENV- and UV-DENV-exposed cells (Table S1). Given that insect cells have a high abundance of PE in their membranes (40–50%), it is interesting that there is a selective requirement for PC (over PE) in DENV-infected mosquito cells [22].

Another group of PLs that were preferentially expressed in DENV-infected cells were the lysophospholipids (LPLs) (Figure 3A and B). These lipids result primarily from the hydrolysis of PC by phospholipase A2-type enzymes (PLA_2_) and represent a PL that is missing an acyl chain [27]–[30]. In DENV-infected cells, there was a preferential up regulation (∼3 fold) of these lipids compared to both UV-DENV and mock controls at the 36 hr time point. This expression was slightly down regulated at the 60 hr time point to about ∼1.5 fold above the mock. In UV-DENV exposed cells, LPLs were undetectable at the 36 hr time point, while at the later time point, they were similar or slightly above the mock levels. As shown in figure 3H, LPLs with C16 acyl chains were up regulated at both time points while there was a selective up regulation of LPLs containing C18 acyl chains only at the early time point (Figure 3H). The C16 LPLs have been implicated in pro-apoptotic signaling pathways. This observation of selected LPL expression presents an attractive hypothesis that chain length differences may dictate specific roles for LPLs during virus infection.

Since LPLs result from the activity of PLA_2_-type enzymes, we investigated whether DENV infected cells displayed a higher activity of PLA_2_ compared to uninfected cells. Utilizing a fluorescent PC substrate (BODIPY-PC) we used mass spectrometry to monitor the hydrolysis of PC to LPC by intracellular PLA_2_. Essentially, we monitored the production of BODIPY-LPC (resulting from PLA_2_ mediated metabolism of PC) during a time course of DENV infection (Figure S1) (method from [31]). The assay indicated that following 24 hr of infection and longer, PLA_2_ activity was elevated in DENV-infected cells compared to the controls, with the highest activity occurring at 48 and 72 hr post-infection. Therefore, the elevated levels of PLA_2_ could be the source of the LPL detected in DENV-infected cells.

#### Sphingolipids

Although originally known as vital components of barrier membranes, sphingolipids are also potent bioactive molecules that regulate cell death, growth, differentiation and intracellular trafficking [32]–[33]. The primary sphingolipids regulated during DENV infection were sphingomyelin (SM) and ceramide (CER). In DENV infected cells, SM was up regulated by ∼2-fold compared to the controls (UV-DENV and mock) and remained elevated at both 36 hr and 60 hr time points (Figure 3A and B). Furthermore, this temporal expression varied depending on the specific molecular species being expressed (Figure 3E and Table S1). UV-DENV exposed cells showed similar levels of SM compared to the mock at the 36 hr time point, but these levels decreased at the later time point. Interestingly, an analog of SM known as ceramide phosphoethanolamine (CER-PE), was preferentially expressed (up regulated by ∼2 fold) in UV-DENV exposed cells at the 36 hr time point (compared to DENV and mock) (Figure 3G). However, this expression level dropped below the mock at the later time point. In comparison, DENV infected cells showed very low expression of this lipid at the early time point (∼0.7 fold compared to mock) and its expression was undetectable at the later time point (Figure 3A and B). This lipid is expressed more abundantly in insect cells rather than in mammalian cells [23]–[24].

Another bioactive sphingolipid, CER, results from either the degradation of SM by sphingomyelinases or *de novo* synthesis through the condensation of palmitate and serine [32], [34]. These lipids were preferentially up regulated in DENV-infected cells at both the 36 and 60 hr time points (∼2-fold compared to the mock). Since SM was also up regulated similarly in DENV infected cells, the rise in CER is possibly the result of *de novo* synthesis rather than SM degradation. UV-DENV-exposed cells showed undetectable CER levels at 36 hr, while at 60 hr the expression was (∼2 fold) above the mock control. Since there was a concurrent decrease in SM in UV-DENV exposed cells at 60 hr, the increased levels of CER in this case could be due to the degradation of SM resulting from increased cellular stress (resulting from incubation of the cells for 60 hr). Similar to other lipid species, CER also showed temporal regulation depending on the molecular species expressed (Figure 3F).

An overall comparison of the PLs regulated at the 36 and 60 hr time points indicated that in all lipid classes that were significantly regulated (Anova p<0.05) during DENV infection, selected molecular species were regulated only at the 36 hr time point while others were regulated at both time points. Representative examples for each lipid class are shown (Figure 3C–H). These observations may represent lipidome differences in cells sustaining early but active DENV replication (36 hr) compared to those experiencing advanced cellular stress (60 hr). It is also interesting to note that any up regulation observed in UV-DENV exposed cells (at either time point) was only in the range of 1–1.5 fold compared to the mock control, while DENV infected cells showed much higher levels of expression.

### Lipidomic analysis of replication complex membranes

**Figure 4:**
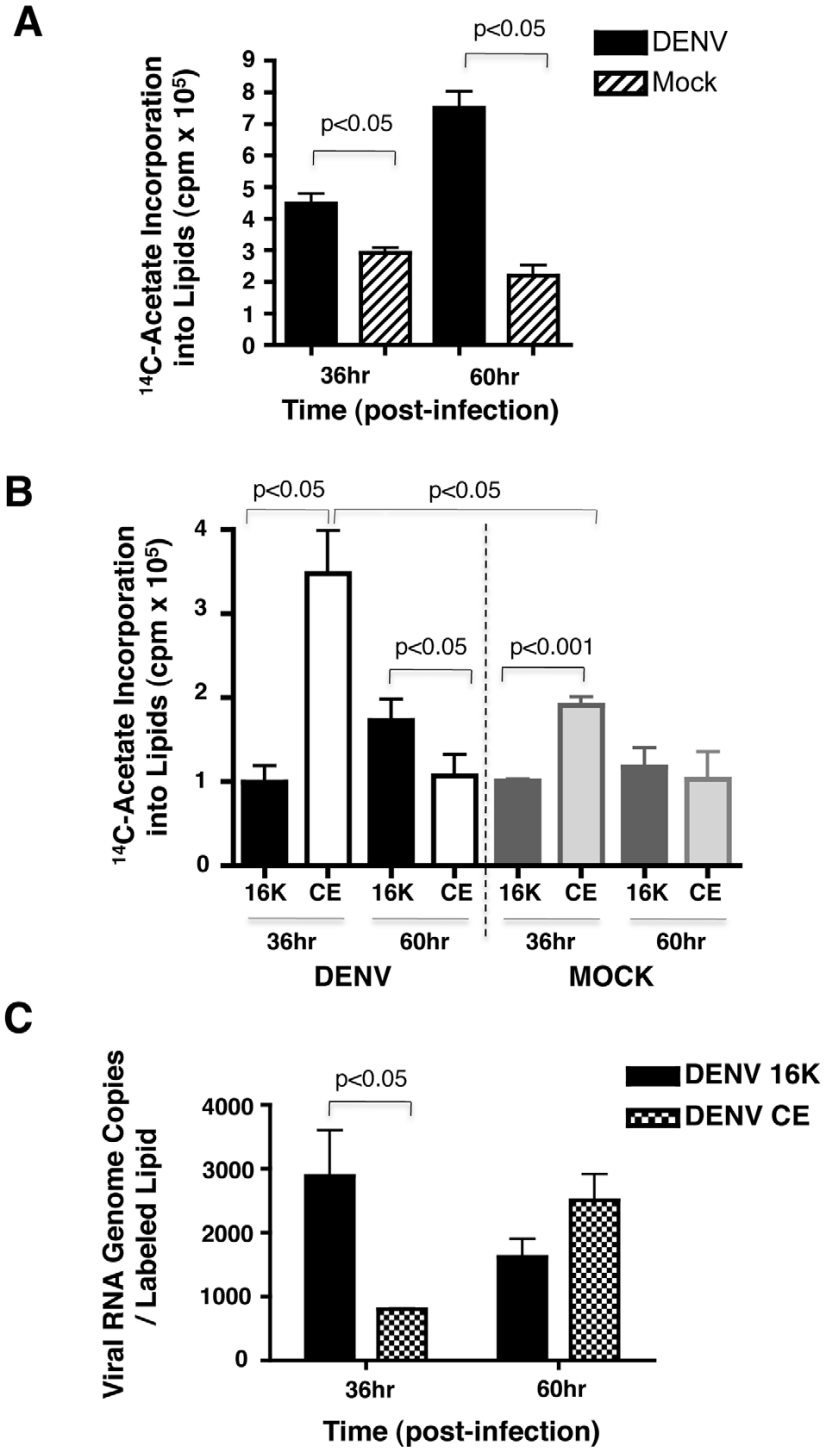
Newly synthesized lipids and viral RNA in subcellular fractions show a dynamic distribution with time of infection. **A.** A pulse-chase analysis of ^14^C-acetate incorporation into newly synthesized lipids. The results show total labeled lipid in post-nuclear supernatants of C6/36 cells infected with DENV for 36 and 60 hr at an MOI of 20. **B.** The same pulse-chase analysis showing ^14^C-acetate incorporation into newly synthesized lipids in subcellular fractions (16K and CE) at 36 and 60 hr post-infection. **C.** The ratio of viral RNA genome copies per labeled lipid in subcellular fractions (described in B) at 36 and 60 hr post-infection. 16K, membrane fraction (pellet) following centrifugation of post-nuclear supernatants at 16, 000× g. CE, cytoplasmic extract following centrifugation of post-nuclear supernatants at 16,000× g. cpm, counts per minute.

In addition to evaluating the lipid composition of whole cells, we also explored the possibility of profiling the lipid repertoire of specific membranes induced by DENV. Given the extensive interconnectivity of the membranes induced in DENV infected cells, isolation of morphologically distinct membranes proved challenging. Therefore, we carried out subcellular fractionation of C6/36 cells and isolated post-nuclear supernatants that were further fractionated to provide a total membrane fraction (16K pellet) enriched in viral replication components and a remaining cytoplasmic extract (CE) [35]. To confirm that the 16K pellet was indeed the membrane fraction enriched in the replication complex components, we used quantitative RT-PCR to measure the ratio of viral RNA to lipid in both 16K and CE fractions. Lipids were quantified by pulse-chase analysis using ^14^C-acetate to label newly synthesized lipids and the results are shown in Figure 4. In post-nuclear supernatants, there was increase in the incorporation of ^14^C-acetate into lipids in the DENV infected cells compared to the mock control (Figure 4A). This increase was observed at both 36 hr and 60 hr post-infection with the latter time point showing a greater difference between DENV and mock. A more detailed analysis of the lipid distribution in the subcellular fractions (16K and CE) is shown in Figure 4B. At the 36 hr time point, the CE fraction was enriched in newly synthesized lipids (compared to the 16K fraction) in both the DENV and mock samples, with DENV showing an almost 2-fold increase compared to mock. This distribution changed at the 60 hr time point where the 16K pellet was more enriched in newly synthesized lipids, however, the fold change (DENV compared to mock) was not as significant.

To identify the primary fraction containing viral induced membranes that included the viral replication complex, we carried out a comparison of the viral RNA genome copies to labeled lipid in the 16K and CE subcellular fractions (Figure 4C). The results indicated that the 16K membrane fraction was enriched (a ∼3-fold increase) in viral RNA (compared to CE) at the 36 hr time point while at the 60 hr time point the CE fraction had slightly more viral RNA per labeled lipid (compared to the 16K fraction).

Based on the ^14^C-acetate labeling studies above, the CE fraction showed an increased amount of newly synthesized lipids (compared to the 16K fraction at the 36 hr time point). In lipid biosynthesis pathways, acetate is a precursor to both phospholipid as well as sterol biosynthesis. Therefore, the labeled lipids (in CE) represent not only phospholipids in membranes, but also cholesterol and triglycerides that form lipid droplets [36]. In this study, lipidomic analyses were carried out on both the 16K and CE subcellular fractions isolated from the 36 hr time point from DENV infected cells, UV-DENV exposed cells and the mock control. The primary lipids observed in the CE fraction were sphingolipids. Cholesterol and triglycerides were not successfully separated using our mass spectrometry analyses and very few phospholipids were observed to be statistically significant (p<0.05) in virus infected cells over the mock control (data not shown).

**Figure 5:**
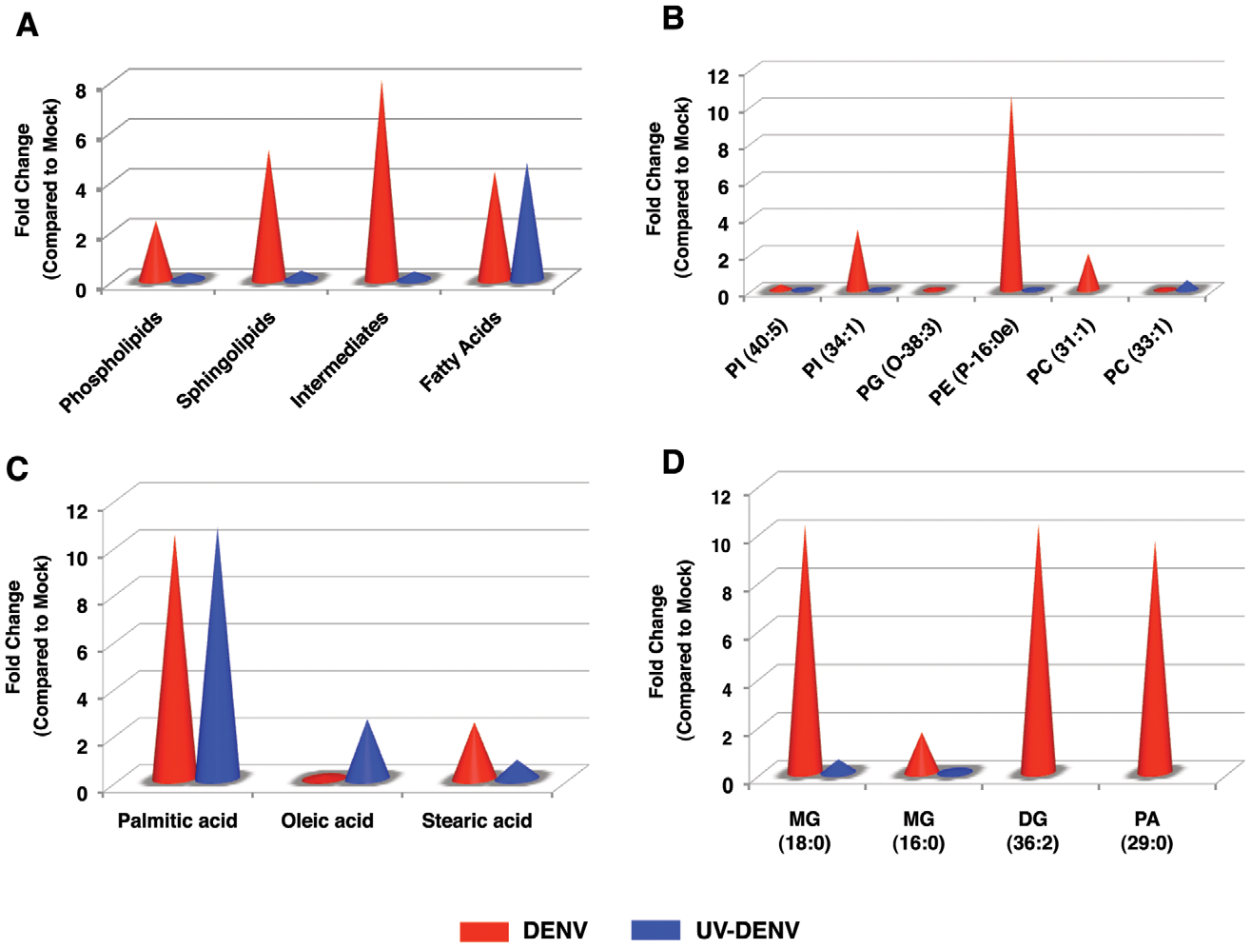
Several lipid classes are differentially regulated in replication complex membranes isolated from DENV infected cells. Panel A shows fold changes for each lipid class significantly (p<0.05) regulated in membrane fractions isolated from DENV infected (red) or UV-DENV exposed (blue) cells compared to the mock control. Infection was carried out using an MOI of 20. Panels B–D show fold changes for individual molecular species within each lipid class that are regulated. PC, phosphatidylcholine; PE, phosphatidylethanolamine; PG, phosphatidylglycerol; MG, monoacylglycerol; DG, diacylglycerol; PA, phosphatidic acid. See supplementary table S2 for a complete list of lipid features detected in this analysis and supplementary figure 1 for a heat map representation of the data. Three replicates were included in the lipidomic analyses.

Therefore, we focused our primary lipidomic analysis on lipids extracted from the 16K pellet at the 36 hr time point. Similar to the whole cell analysis, DENV infected cells displayed a unique expression profile compared to UV-DENV and mock infected cells (Figure S2). Over 85% of the metabolites detected were significantly regulated in virus-infected cells compared to the controls. Analysis of the membrane fraction also highlighted several lipid species not detected in our whole cell analysis. An overall fold change (compared to mock) of the significantly (p<0.05) expressed lipid classes in the membrane fraction is shown in Figure 5. For a complete list of differentially expressed lipids see Table S2.

#### Phospholipids

There was an up regulation of phospholipids, sphingolipids and several bioactive intermediates in DENV infected cells compared to UV-DENV and mock controls (Figure 5A). Amongst the specific phospholipids expressed, PE was selectively up regulated in membrane fractions isolated from DENV infected cells (Figure 5B). However, the primary PE molecular species up regulated was a lysophospholipid (PE P-16:0e). Both DENV and UV-DENV exposed cells showed similar expression of fatty acids (Figure 5A). However, the individual molecular species expressed differed between the two viruses (Figure 5C). For instance, stearic acid was up regulated in DENV infected cells while oleic acid was up regulated in UV-DENV exposed cells. Palmitic acid had a similar level of expression in both DENV and UV-DENV exposed cells. Several bioactive intermediates such as monoacylglycerol (MG), diacylglycerol (DG) and phosphatidic acid (PA) were also prominently up regulated in DENV infected cells compared to the controls (Figure 5D).

#### Sphingolipids

Although the Blygh and Dyer method [37] utilized for lipidomic analyses of whole cells or membrane fractions detected the expression of sphingolipids, Merrill et al optimized a protocol dedicated to analyzing sphingolipids without the interference from co-purifying PLs [38]. Utilizing this protocol, we carried out mass spectrometry analysis of these bioactive lipids from mosquito cells fractionated to provide three subcellular fractions: nuclear (N), cytoplasmic (CE) and membrane (16K). Similar to the above analyses, UV-DENV and mock infected cells were included as controls. The specific sphingolipids monitored included CER, SM, monohexosylceramide (GlcCER) and dihexosylceramide (GalCER).

**Figure 6:**
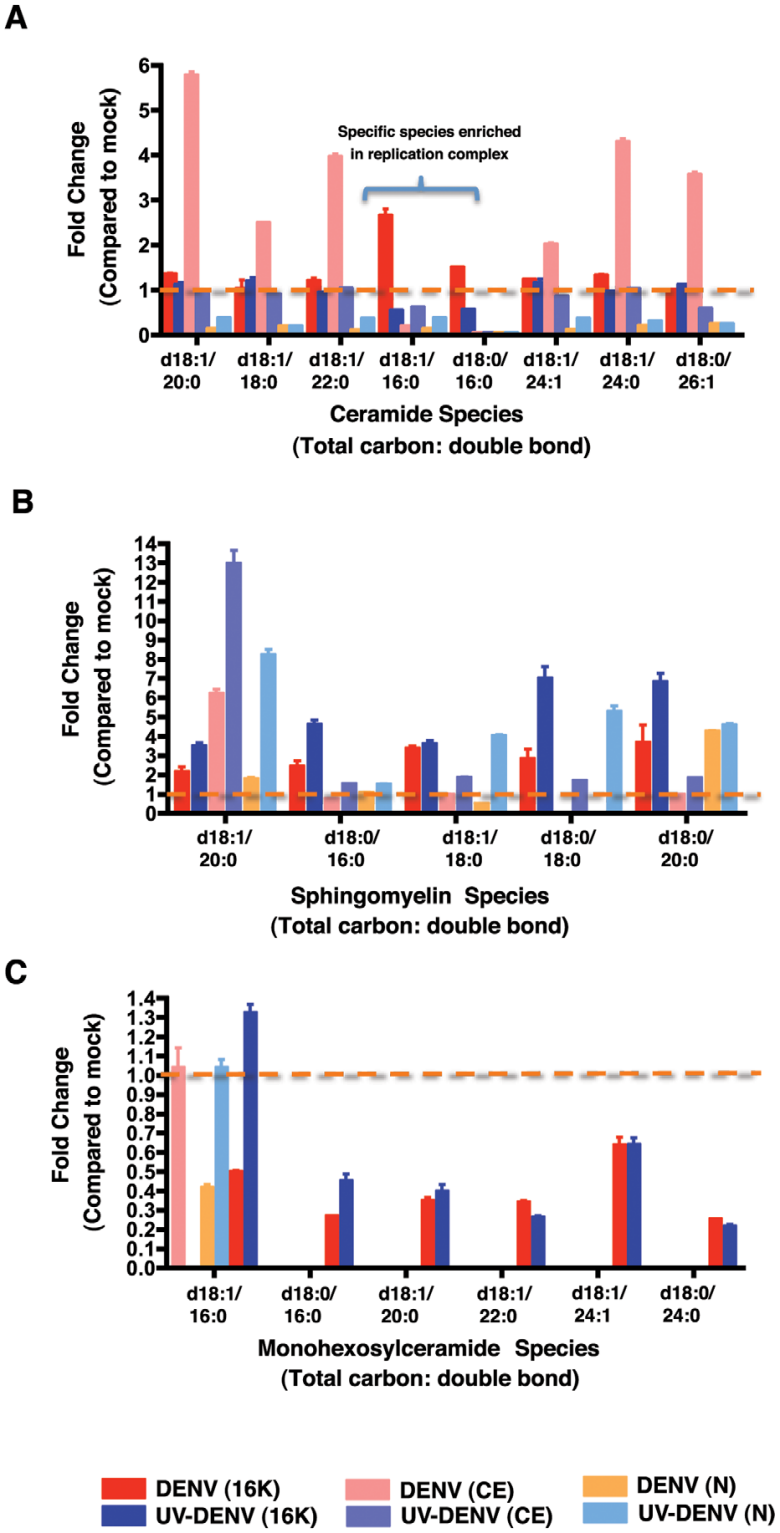
Bioactive sphingolipid species are differentially regulated in replication complex membranes isolated from DENV-infected mosquito cells. Multiple Reaction Monitoring (MRM) analysis of sphingolipids species differentially regulated in DENV-infected cells (MOI 20) or UV-DENV exposed cells compared to the mock control (see also supplementary table S3). The data represent fold changes observed in three subcellular fractions that were analyzed in this study; 16K, replication complex membranes; CE, cytoplasmic extracts following removal of replication complex membranes and nuclei; N, nuclear fraction. Panels A–C represent ceramide, sphingomyelin and monohexosylceramide species respectively. The dashed line highlights values equal to the mock. The data represent three independent experiments. The error bars represent standard deviation of the mean.

As shown in Figure 6A, CER levels in C6/36 cells showed clear elevation in DENV-infected cells compared to mock-infected or UV-DENV exposed cells. Comparison of the different subcellular fractions indicated that the elevated CER was primarily concentrated in the CE fraction with the exception of two species (d18:1/16:0 and d18:0/16:0) which were enriched in the 16K pellet. These two species consist of the most abundant fatty acids observed in cells. The analysis of SM levels in mosquito cells (Figure 6B) indicated that virus exposed cells have a higher level of SM compared to mock-infected cells. This is most evident in the 16K pellet with the exception of the most abundant species (d18:1/20:0) that showed the highest levels in the CE. Comparison of infectious DENV to UV-DENV exposed cells indicates that the latter which is incapable of replication maintained higher levels of SM overall.

GlcCER were primarily detected in the 16K pellet of both DENV infected and UV-DENV exposed cells (Figure 6C). However, the overall levels were down-regulated compared to the mock control with the exception of d18:1/16:0, which showed slight elevation in the UV-DENV exposed cells. Dihexosylceramides were not detected to significant levels in any of the samples.

### Analysis of lipid re-distribution upon C75 treatment of mosquito cells

The FAS inhibitor, C75 is a potent inhibitor of DENV replication in both mosquito (Figure 1) and mammalian cells [20]. Cells treated with non-cytotoxic concentrations of C75 (<50 µM) induce an environment that has redistributed its lipid repertoire to support cell survival in the absence of FAS activity, and yet, this environment does not support viral replication. To determine the basis for this exclusion of virus replication, we profiled the lipidome of cells treated with 25 µM C75. Membrane fractions isolated from DENV infected and mock control cells in the presence or absence of C75 were profiled. A hierarchical clustering analysis of the lipidomic data (Figure S3) indicated that the lipid environment of DENV infected cells treated with C75 clustered very closely to the mock C75 treated control and was furthest from DENV infected cells suggesting that the two represent significantly different environments. A comparative analysis of the lipid species expressed in the two environments indicated that a majority of the lipids that were up regulated in DENV infected cells were down regulated upon treatment of those cells with C75.

**Figure 7:**
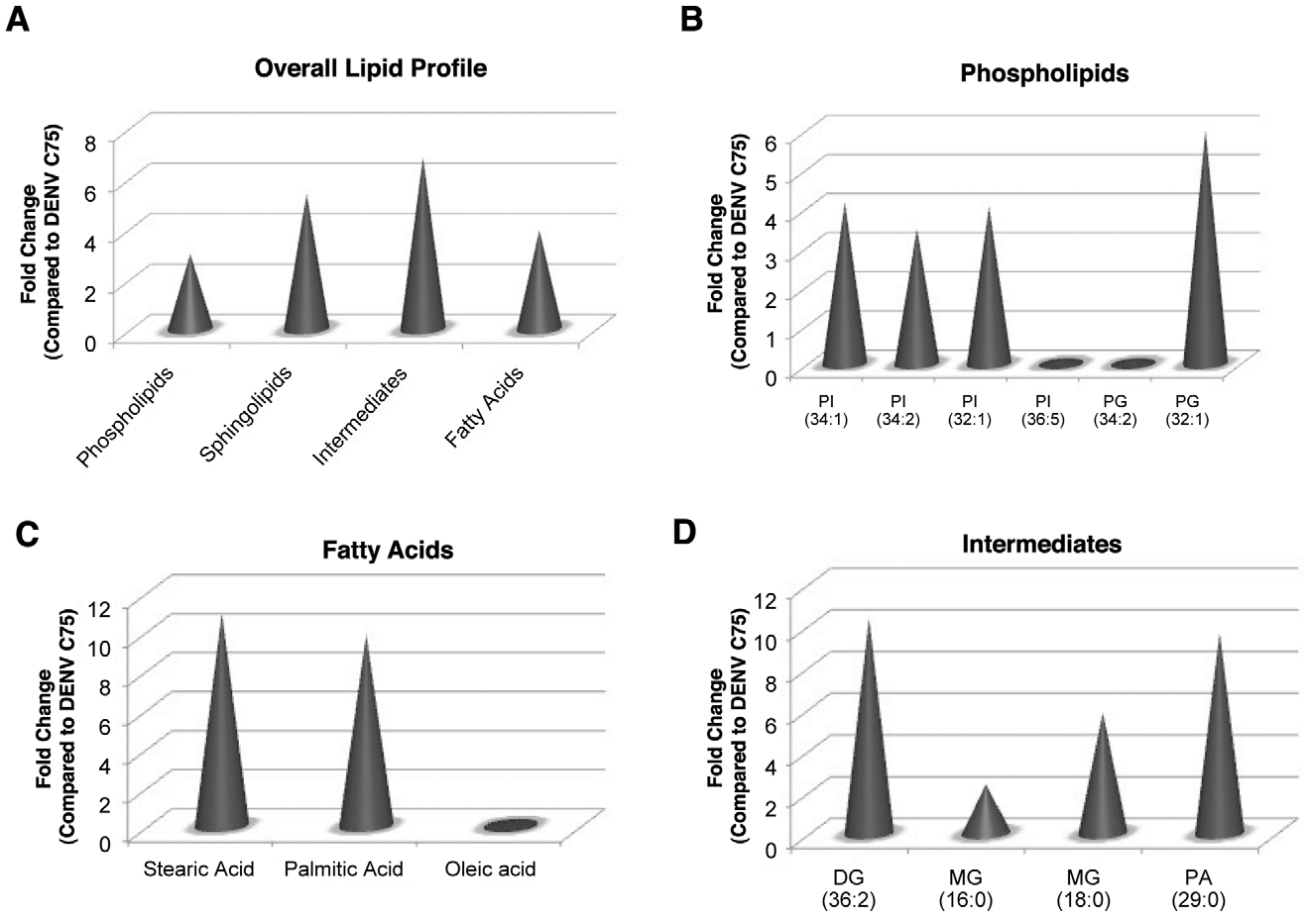
The Lipid repertoire of DENV infected mosquito cells is unfavorable for replication in the presence of C75. The specific lipid classes differentially regulated by C75 treatment of cells are shown. Panel A represents the fold changes of lipid classes expressed in DENV infected cells (MOI 3) compared to C75 treated DENV infected cells. Panels B–D show fold changes for individual molecular species within each lipid class that are regulated. PI, phosphatidylinositol; PG, phosphatidylglycerol; MG, monoacylglycerol; DG, diacylglycerol; PA, phosphatidic acid. See supplementary table S3 for a complete list of lipid features detected in this analysis and supplementary figure 2 for a heat map representation of the data. Three replicates were included in the lipidomic analyses.

Amongst the phospholipids, several phosphatidylinositol species were up regulated 3–4 fold in the untreated DENV infected cells compared to the C75 treated cells (Figure 7B). However, none of these PI species were phosphorylated. A single species of PG was also up regulated by over 5 fold in the untreated cells. Since C75 disrupts the formation of lipids down stream of FAS, several fatty acids (stearic and palmitic acid) were down regulated in the drug treated cells. An enrichment of these fatty acids was observed in the untreated cells upon comparison with C75 treated cells (Figure 7C). In contrast, as observed in the previous comparison of DENV infected cells to uninfected cells (Figure 5), oleic acid was down-regulated in untreated DENV infected cells and therefore, showed enrichment in C75 treated cells. Interestingly, C75 treated cells also showed a down regulation of metabolic intermediates such as mono- and diacylglycerol as well as phosphatidic acid. Enrichment of these lipids was observed in untreated DENV infected cells compared to C75 treated cells (Figure 7D). In addition to the phospholipids, *de novo* synthesis of sphingolipids was also disrupted by C75. Key intermediates in this pathway such as N-palmitoylesphingosine and N-stearoylesphingosine were down regulated upon treatment of cells with C75 and up regulated in untreated DENV infected cells (Figure 7A and Table S3).

## Discussion

The architecture of biological membranes is defined by the composition and distribution of lipids and proteins in the bilayer. Optimizing this composition and distribution to facilitate the formation of virus-induced intracellular membrane structures has to be a requirement for efficient DENV replication. Previously we showed that FAS, the rate-limiting enzyme in lipid biosynthesis was both recruited and activated during DENV replication in human cells [20]. In this study we demonstrated that this requirement for FAS activity was also conserved in the mosquito vector-derived cells. Furthermore, inhibition of FAS activity seemed to be most effective between 4–12 hr post-infection suggesting an early requirement for FAS activity during the replication cycle.

**Figure 8:**
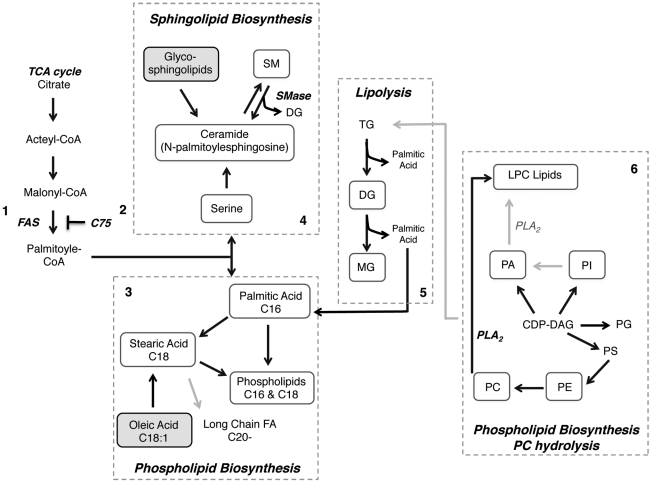
Dengue virus infection perturbs lipid homestasis in infected mosquito cells. The lipidomic analyses of dengue virus infected C6/36 mosquito cells suggest several metabolic pathways that may be significantly up regulated during infection. The grey dashed line highlights specific pathways of interest. Black arrows highlight reactions suggested by the lipidomic data and grey arrows represent reactions not observed in the data. Metabolites highlighted in boxes (solid line) are up regulated (white) or down regulated (grey) in DENV infected mosquito cells. **1.** Through the recruitment and activation of FAS, DENV stimulates *de novo* phospholipid biosynthesis in the replication complex [20]. **2.** Inhibition of this process with C75 disrupts the cellular lipid repertoire in mosquito cells to be unfavorable for virus replication. **3.** The lipidomic analyses reveal an up regulation of fatty acids such as palmitic (C16) and stearic (C18) acid. These fatty acids are intermediates in the biosynthesis of phospholipids, which is up regulated during DENV infection. Interestingly, in DENV infected cells the prevalent phospholipids primarily consist of C16 and C18 unsaturated acyl chains. Very long chain fatty acids are not significantly up regulated during infection. **4.** FAS activity also stimulates *de novo* sphingolipid biosynthesis. In the lipidomic analyses, the up regulation of intermediates such as N-palmitoylesphingosine suggests sphingolipid biosynthesis is activated during DENV infection. Specifically, SM and CER are enriched in DENV infected cells. Alternately, the up regulation in CER (and DG) during infection could result from the degradation of SM through the activity of sphingomyelinases (Smase). The resulting CER and DG could be redirected into several signaling pathways or be utilized for *de novo* phospholipid biosynthesis. The glycopshingolipids, GlcCER and GalCER are down regulated during DENV infection, which suggest that they are catabolized to produce CER. **5.** Lipidomic analyses also suggest the up regulation of triacylglycerol catabolism (Lipolysis) in DENV infected cells. This pathway results in the generation of MG, DG and palmitic acid. These intermediates are all up regulated in DENV infected cells and could be utilized for downstream signaling or *de novo* phospholipid biosynthesis. It has also been shown that TG catabolism is necessary for mitochondrial β-oxidation during DENV infection [50]. **6.** Elevated levels of LPC in DENV infected cells also suggest activation of PC hydrolysis by PLA_2_. This enzyme is activated during DENV infection. The elevated levels of other phospholipids such as PA, PI, PE, PG as well as PC suggest that the CDG-DG pathway for phospholipid biosynthesis could also be activated. FAS, fatty acid synthase; DENV, dengue virus; C75, inhibitor of FAS; SM, sphingomyelin; CER, ceramide; MG, monoacylglycerol; DG, diacylglycerol; TG, triacylglycerol; LPC, lysophosphatidylcholine; PLA_2_, phospholipase A_2_; PA, phosphatidic acid; PI, phosphatidylinositol; PE, phosphatidylethanolamine; PG, phosphatidylglycerol; PC, phosphatidylcholine.

As a next step in investigating the role of lipids in DENV induced membrane expansion we used high-resolution mass spectrometry to profile the lipid composition of DENV infected mosquito cells. We observed a very distinct segregation in the lipid composition between DENV infected and uninfected cells. Specific lipid changes that occurred upon virus binding and entry alone were also identified. In DENV infected cells, there was a selective enrichment of lipids that have characteristic functions in influencing membrane structure as well as those that have potent signaling functions. Among these lipids were bioactive sphingolipids such as sphingomyelin and ceramide, lysophospholipids and several intermediates such as mono- and diacylglycerol and phosphatidic acid. The phospholipids that were enriched in DENV infected cells were primarily unsaturated. There was also evidence for the up regulation of *de novo* phospholipid and sphingolipid biosynthesis as well as triacylglyceride metabolism (Figure 8). Furthermore, we identified a unique lipid environment that supported cell survival but did not support DENV replication. This environment was created by the addition of C75, an inhibitor of fatty acid synthase, the rate-limiting step in phospholipid biosynthesis.

Analysis of the whole cell lipidome indicated that unsaturated PC was the most up regulated phospholipid in DENV infected cells. Membranes that are similar or derived from the ER are enriched in unsaturated lipids, primarily PC [39]. Although DENV-induced membranes in mammalian systems are ER-derived, it is unknown if the same is true in mosquito cells. While PC is known to form planar membranes, unsaturated PC induces membrane curvature which may be important for maintaining the highly curved membranes observed in DENV-infected cells [40]. PC enriched membranes are also more fluidic compared to the rigid membranes enriched in SM and cholesterol, which may be a feature attractive to DENV infection [41].

Bioactive sphingolipids such as SM and CER are also key lipids that were up regulated during DENV infection. In cells exposed to replication competent virus, CER was up regulated in both the whole cell lipidome as well as replication complex membranes. This is either a cellular response to virus infection, or a direct need for CER in the virus replication cycle. The latter hypothesis is particularly attractive considering the enrichment of specific molecular species of CER in the 16K pellet. CER is a cone-shaped molecule that promotes inward budding of membranes (negative curvature) [42]. Some DENV-induced membranes show a double membrane morphology indicating that negative curvature modifying lipids such as CER may be active in their formation.

As a second messenger, CER can also induce apoptosis and autophagy [34]. However in mosquito cells, DENV maintains a persistent infection and antagonizes apoptotic pathways [19], [43]. Therefore, CER up regulation may not be due to the prevalence of an apoptotic response but may be utilized to up regulate autophagy during DENV infection. It has been shown in mammalian cells that autophagy is up regulated and necessary for DENV replication. The prevalence of several intermediates in CER biogenesis, including N-palmitoylsphingosine and diacylglycerol, suggest that the observed CER could result from either de novo synthesis by serine palmitoyltransferase (SPTLC) in the ER or catabolism of SM by sphingomyelinases (SMases) at the plasma membrane [44]. The lipidomic analyses also indicated a down regulation of GlcCER, which may suggest that a salvage pathway that metabolizes GlcCER to CER may also be active.

Inverted cone-shaped lipids such as LPLs were also up regulated during DENV infection. Primarily, LPC was up regulated in the whole cell analysis. It results from the hydrolysis of PC by PLA_2_ [45]. We have shown that this enzyme is activated during DENV infection. Many other viruses have also shown a dependency for PLA_2_ in their life cycle [31], [46]. Due to its inverted cone-shaped structure, asymmetric incorporation of LPC into membranes causes positive curvature and induces vesicle fission and budding [47]. Structural analyses of DENV-induced membrane structures in mosquito cells indicate the presence of highly curved membranes and smaller vesicles [18]–[19]. Therefore, vesiculation through fission and budding as well as enrichment of molecules that increase membrane curvature may be an underlying mechanism for forming these membrane structures.

LPC also increases the permeability of membranes [48]. This is an attractive concept given that DENV replication complexes are encased within membrane barriers that need to facilitate the exchange of components to and from the cytosol for genome replication and virus assembly. Therefore, leaky membranes may be favored in DENV-infected cells through the selective incorporation of molecules such as LPC. Another observation that gives credence to this hypothesis is that the transmembrane permeabilizing effect of LPC is synergistically enhanced in the presence of palmitic acid, which is also up regulated in DENV-infected cells. As a signaling molecule, LPC is both a pro-survival as well as an inflammatory molecule [46]. Therefore, its expression in DENV infected cells could be a cellular response to viral infection.

### Evidence for lipogenesis

It has been previously reported that *de novo* lipid biosynthesis was up regulated during DENV infection [20]. Consistent with these observations, several primary fatty acids (palmitic, stearic and oleic acid) involved in the biosynthesis of higher order PLs were detected in our lipidomic analyses. Palmitic acid is the first fatty acid of the lipogenesis pathway and stearic acid (C18:0), is immediately downstream of palmitic acid. They are both up regulated in DENV infected cells. The latter is the precursor to the biosynthesis of oleic acid (C18:1) or long chain fatty acids (with acyl chains of C20 or greater). Interestingly, since oleic acid is down regulated in DENV-infected cells stearic acid may be stimulating long chain FA biosynthesis. However, a majority of the PLs expressed in DENV infected cells have C16- or C18-acyl chains with varying degrees of unsaturation. Therefore, it seems that desaturation of palmitic and stearic acid rather than elongation may be the chosen pathway for lipogenesis during DENV infection.

### Evidence for lipolysis

Several intermediates in lipid catabolism were also detected in the replication complex membranes (palmitic acid, MG and DG). Analysis of these intermediates suggested the up regulation of pathways for PC hydrolysis and triacylglycerol (TG) metabolism. The latter pathway is responsible for regulating lipid homeostasis and energy production in the cell through the metabolism of lipid droplets [49]. Through the action of lipases the degradation of lipid droplets results in the release of palmitic acid, DG and MG [36]. These metabolic products are substrates for mitochondrial β-oxidation as well as intermediates (fatty acids) in the synthesis of new lipids. DENV infected cells show a high prevalence of these species compared to mock infected cells.

While several acidic phospholipid species were detected in the 16K membrane fraction very few were significantly regulated during infection. Previous studies in mammalian cells have indicated the importance of acidic lipids in DENV infection [46], [50]. These negatively charged lipids are also implicated in influencing membrane structure.

The most striking results were obtained by the comparison of DENV infected cells in the presence and absence of the FAS inhibitor, C75. It is well established that treatment of cells with this inhibitor disrupts *de novo* phospholipid biosynthesis [51]. To circumvent this inhibition, the cell redistributes its lipid repertoire to ensure cell survival. However, this redistribution does not support DENV replication. A comparison of the different lipid environments indicated that many of the lipids that were up regulated in DENV infected cells compared to cells treated with C75, were similar to those previously observed when infected cells were compared to mock (uninfected) controls. Therefore, comparison of DENV infected cells to two different controls (uninfected cells and C75 treated infected cells) highlighted the same lipids as being up regulated during an active DENV infection.

In these studies we utilized UV-DENV as a control to identify lipid metabolic changes that occurred upon virus binding and entry alone. This virus is incapable of replication but immunofluorescence microscopy with anti-NS3 antibodies have indicated that there is a very low level of translation that occurs within the first 24 hr (data not shown). Analysis of the lipidome of UV-DENV exposed cells showed a significant difference in the phospholipid and sphingolipid content in comparison to DENV infected cells and the mock control. It is possible that binding, membrane fusion and entry alone, or the expression of viral proteins from the incoming viral RNA triggers the activation of lipases or lipid transport mechanisms that result in the differences observed in the lipidome of the cells. This has been shown for other viruses. Jan et al. have shown that binding and entry alone of Sindbis virus triggered the activation of sphingomyelinases that degraded membrane bound SM to Cer [52]. It has also been shown that viruses induced apoptotic-signaling cascades upon binding and entry alone [53]–[55]. These cascades may either result from or induce lipolysis or lipid transfer between compartments in the cell.

The most pronounced changes are those observed in the 16K pellet analyses where a significant decrease in phospholipids, sphingolipids and lipid intermediates are observed (compared to mock cells, Figure 5A). A comparison of SM and CER in the whole cell analysis at the 36 hr and 60 hr time points (Figure 1A and 1B) indicated a reduction in SM with a corresponding increase in CER with time. This could be due to a similar scenario to SINV where a non-replicating UV-DENV is capable of inducing lipid conversion in membranes [52]. Therefore, SM and other sphingolipids may be converted to CER that is transported away from the 16K membrane fraction to other locations in the cell. This is also evident from the increased content of fatty acids compared to mock cells that may result from the degradation of lipids. UV-DENV exposed cells do not show significant regulation of MG or DG, but show an up regulation of palmitic acid (C16:0). Virus binding and entry alone could stimulate lipid droplet hydrolysis (catabolism of TG) resulting in the release of palmitic acid. These studies have shown that UV-DENV could be a versatile tool to pursue mechanistic studies on the metabolic pertabations that occur upon virus binding, membrane fusion, and entry.

In summary, using high-resolution mass spectrometry, we have determined that DENV drastically alters the lipid profile of infected cells. Specifically, DENV infection elevates the expression of lipids that have the capacity to change the physical properties of the bilayer such as bilayer curvature, permeability, and the recruitment and assembly of protein complexes in the membrane. Several of the identified molecules also function as bioactive messengers that control signaling and membrane trafficking pathways in the cells. They represent molecules that result from the activation of cellular stress pathways that respond to viral infections. Based on these findings, the next steps will be to investigate the mechanisms of how these lipid species play a role in DENV replication, as well as identify the control points in these pathways that may be influenced by viral gene products.

## Methods and Materials

### Cell culture and virus infections

C6/36 (*Aedes albopictus*) cells (ATCC) were maintained in Minimal Essential Medium (MEM) supplemented with glutamine (2 mM), non-essential amino acids, 25 mM Hepes and 10% heat inactivated fetal calf serum. Dengue virus 2, strain 16681 (obtained from Richard Kinney, CDC. Ft. Collins) stocks were amplified in C6/36 cells in MEM (supplemented as above) and 2% heat inactivated fetal calf serum. UV-inactivated DENV was obtained by exposing the same virus stock (used for infection) to UV-light for 3 hr and then conformation of inactivation by two blind passages of the virus on cells for 60 hr per passage. Lack of infectivity was confirmed by plaque assay and immunofluorescence assay.

### Lipidomic sample preparation and mass spectrometry analyses of whole cells

#### Sample preparation

For infection, ∼5×10^8^ C6/36 cells were infected with DENV at a MOI of 20 at 30°C. Infection of all cells (required for lipidomic analyses) was confirmed by immunofluorescence analyses. Each experiment included three biological replicates. Following infection, cells were harvested at 36 and 60 hr post-infection, pelleted and resuspended in 100 µl of 100 mM ammonium bicarbonate. Lipids were extracted from an equal number of cells using a modified Bligh and Dyer protocol. Briefly, a mixture of 2∶1 Chloroform∶methanol, 0.1% acetic acid and 0.01% butylated hydroxy toluene (BHT) were added to the cell suspension in ammonium bicarbonate such that there was a 4∶1 ratio of organic solvent to cells. Following lipid extraction, the organic phase was separated from the aqueous phase by centrifugation and dried down under a N_2_ stream in low retention microfuge tubes (Fisher). The dried lipids were resuspended in 75 ml of methanol and vortexed for 10 s. The samples were then centrifuged at 13,400× g for 5 min to remove any particulates.

#### Liquid chromatography/mass spectrometry

Lipid molecular species were profiled using a dual-column nanocapillary LC system equipped with 75 µm×65 cm columns, each packed with 3 µm Jupiter particles (Phenomenex, Torrance, CA). The mobile phases were (A) 10 mM ammonium acetate in 50∶50 water/methanol (v/v) and (B) 10 mM ammonium acetate in 50∶50 methanol/acetonitrile (v/v). The LC system was equilibrated at 10,000 psi with mobile phase A prior to injecting 0.4 µL of sample. Exponential gradient elution was initiated 50 min after sample loading with an initial column flow of ∼300 nL/min. After 90 min of gradient separation, the mobile phase mixer was purged with 3 mL of mobile phase B, followed by a 5 min column wash. Finally, the mobile phase mixer was purged with 10 mL of mobile phase A, which represented the end of one separation cycle. While gradient elution was performed on one column, the other column was equilibrated with mobile phase A. The LC system was coupled to a hybrid linear ion-trap-Orbitrap mass spectrometer (ThermoFisher, San Jose, CA), and the capillary temperature and electrospray voltage were 200°C and +2.2 kV, respectively. The Orbitrap was used as the mass analyzer during MS survey scans over the m/z range 200–2000 with a duty cycle of ∼1.2 s. Data-dependant MS/MS was performed in the LTQ for the top 5 ions, with a normalized collision energy of ∼35%. Dynamic exclusion in the LTQ during data-dependant MS/MS experiments was enabled as follows: repeat count of 2, repeat duration of 30 s, exclusion list size of 250, and exclusion duration of 60 s.

#### Data processing

LC-MS datasets, defined as the data obtained from a single LC-MS analysis, were processed using the PRISM Data Analysis system [56], a series of software tools freely available at http://ncrr.pnl.gov/software/ and developed in-house. The first step involved deisotoping of the raw MS data to give the monoisotopic mass, charge state, and intensity of the major peaks in each mass spectrum using Decon2LS [16]. The data were next examined in a 2-D fashion using MultiAlign to identify groups of mass spectral peaks that were observed in sequential spectra using an algorithm [57] that computes a Euclidean distance in n-dimensional space for combinations of peaks. Each group, generally ascribed to one detected species and referred to as a “feature”, has a median monoisotopic mass, central normalized elution time (NET), and abundance estimate computed by summing the intensities of the MS peaks that comprise the entire LC-MS feature. LC-MS features were then chromatographically aligned across all datasets using the LCMSWARP algorithm [58] in MultiAlign, and the lipid identities of detected features were initially determined by comparing their measured monoisotopic masses and NETs to calculated monoisotopic masses and observed NETs for lipids in an AMT tag database [59] within search tolerances of ±3 ppm and ±0.03 NET for monoisotopic mass and elution time, respectively. Subsequent identifications of lipid features that did not match to entries in the AMT tag database were made by searching their molecular weights against entries in the Lipid MAPS database followed by manual confirmation based on MS/MS spectra.

#### Statistical analysis of processed data

Following chromatographic alignment and database matching, the abundances of all detected features (both AMT tag database matched and unmatched) were loaded into DAnTE [60] for statistical analysis. Feature abundances were transformed to log2 scale then subjected to central tendency normalization [61]. Comparative data analysis was then performed and statistically significant differences between the lipid profiles of the samples were determined using ANOVA. Partial least-squares (PLS) [62] analysis was also performed using the data matrices containing either AMT tag database matched features alone or all features (both database matched and unmatched).

### Pulse-chase analyses and quantitative RT-PCR

Virus infection of C6/36 cells was carried out as described for the whole cell analyses. To label newly synthesized lipids, Acetic Acid, Sodium Salt, [1-^14^C] (Perkin Elmer) was added to cell supernatants at 8 hr post-infection. At 12 hr post-infection, the label containing supernatant was removed and new medium was added to the cells. At 36 and 60 hr post-infection, cells were harvested by trypsinization and washed with buffer A (35 mM Hepes, pH 7.4, 146 mM NaCl, 11 mM glucose). Cells were then osmotically shocked in buffer B (20 mM Hepes, pH 7.4, 10 mM KCl, 1.5 mM MgOAc and 1 mM DTT) for 30 min on ice, homogenized and centrifuged at 1500× g, at 4°C for 5 min to remove nuclei. Post-nuclear supernatants were centrifuged at 16000× g for 30 min at 4°C to obtain a membrane pellet (referred to as the 16K pellet). The supernatant post nuclear and membrane fractionation is referred to as the cytoplasmic extract (CE). Total RNA was extracted from each fraction (16K and CE) using Trizol reagent (Sigma) according to the manufacturer's instructions. Following RNA extraction, total lipids were extracted using the Bligh and Dyer method mentioned above and counted using a Beckman LS 6000 scintillation counter. The viral RNA in each fraction was measured using the SuperScript III Platinum SYBR Green One-Step qPCR Kit with ROX and DENV specific primers; (forward) 5′ ACAAGTCGAACAACCTGGTCCAT 3′ and (reverse) 5′ GCCGCACCATTGGTCTTCTC 3′ on a Applied Biosystems 7300 Real Time PCR machine. The labeled lipid in subcellular fractions was standardized to the total RNA isolated from each fraction prior to determining the viral RNA genome copies per labeled lipid in subcellular fractions.

### Lipidomic sample preparation and mass spectrometry analyses of replication complex membranes

#### Sample preparation

For profiling the lipidome of the replication complex membranes, lipids were extracted from the 16K pellet using the same modified Blygh and Dyer protocol described above.

#### Liquid chromatography/mass spectrometry profiling analysis of lipid content

A LTQ Orbitrap XL instrument (Thermo Fisher Scientific San Jose, CA) was used for the analysis. It was coupled to an Agilent 1100 series LC (Agilent Technologies, Santa Clara, CA) equipped with a micro well plate auto sampler and binary pumping device. Reverse phase liquid chromatography was used to analyze the samples. An Agilent Eclipse XDB-C8 column with 2.1×150 mm, 3.5 µm dimensions was used for the separation. Solvent A consisted of water +0.1% piperidine. Solvent B contained acetonitrile ∶ methanol (50∶50 v/v) +0.1% piperidine. The flow rate was 300 µL/minute. A volume of 10 µL was loaded onto the column. The gradient was as follows: time 0 minutes, 50% B; time 25 minutes, 95% B; time 45 minutes, 95% B; time 50 minutes, 50% B; time 60 minutes, 50% B. The MS analysis used negative polarity electrospray ionization. The source voltage was 4.0 kV, source current 100 µA, capillary voltage −30.0 V, tube lens voltage −100.0 V. The capillary temperature was 200°C, sheath gas flow was 35, auxiliary and sweep gas were both set to 0. Data were acquired using data dependent scanning mode. FTMS resolution of 60,000 with a mass range of 70–1200 was used for full scan analysis and the ITMS was used for MS/MS data acquisition. The top three most intense ions were acquired with a minimum signal of 500, isolation width of 2, normalized collision energy of 35, default charge state of 1, activation Q of 0.250, and an activation time of 30.0. The samples were evaluated with Thermo XCalibur software (version 2.1.0) and downstream alignment done with an in-house data processing package called Omics Discovery Pipeline [63], [64].

#### Liquid chromatography/mass spectrometry for targeted sphingolipid analysis

The method was slightly modified from [65]. Briefly, each sample was extracted according to published protocol then dried and reconstituted in 100 µL of methanol ∶ water ∶ formic acid (74∶25∶1) containing 5 mM ammonium formate. An Agilent 6400 QQQ (Agilent Technologies, Santa Clara, CA) was used for analysis coupled to an 1100 Series LC equipped with HPLC Chip interface (Agilent Technologies Santa Clara, CA). Solvent A consisted of methanol ∶ water ∶ formic acid (74∶25∶1) containing 5 mM ammonium formate and solvent B methanol ∶ formic acid (99∶1) containing 5 mM ammonium formate. The gradient was as follows: time 0 minutes, 20% B; time 1 minute, 20% B; time 3 minutes, 100% B; time 23 minutes, 100% B; time 24 minutes, 20% B; time 30 minutes, 20% B. The flow rate was 0.4 µL/min. Data were acquired in MRM mode with transitions and collision energy according to Merrill et al [65] with the following source conditions: gas temperature 300°C, gas flow 4 L/minute, capillary voltage 1900 V. The fragmentor voltage was set to 180 V in all cases. Data were processed with Agilent Mass Hunter software version B03.01.

### C75 inhibitor studies

C6/36 cells were infected with DENV at an MOI of 3. Following adsorption, virus was removed and the cells were washed and overlayed with media containing varying concentrations of C75 [51]. The vehicle for C75 was ethanol. Virus supernatants were harvested at 24 hr post-infection and virus titer assayed by plaque assay. Cytotoxicity of C75 was simultaneously assayed using the Quick Cell Proliferation Kit (Abcam). **Time of addition of C75:** C6/36 cells were infected with DENV as described above. At the indicated time points, 6.3 µM C75 was added to the cells. Cells were harvested at 24 hr post-infection and the amount of released virus was determined by plaque assay. For the lipidomic studies, cells were infected with DENV (as described above) or left uninfected. Following adsorption of the virus at room temperature for 2 hr, C75 was added to the media in the overlay, and cells were incubated at 30°C for 36 hr. Sample preparation, lipid extraction and mass spectrometry analyses were carried out as described above for the fractionated (16K pellet) samples.

### Phospholipase A (PLA) analyses

Phospholipase A2 activity was monitored using a Red/Green BODIPY PC-A2 substrate (Invitrogen) using a similar method as described in [31]. C6/36 cells were infected with DENV at an MOI = 3 in 6-well plates. Following virus adsorption, the cells were overlayed with MEM (supplemented as above) and 10% heat inactivated fetal calf serum (2 ml/well). At the indicated time points, the media were removed, and new media (1 ml/well) containing 6 µM of the fluorogenic phospholipase A substrate (BODIPY-PC) were added to the cells. The cells were incubated at 30°C for 30 min. Following incubation, media was removed and the cells were washed with 1× PBS. The lipids were then extracted using butanol-1 (1 ml/well). The aqueous fraction was discarded and the lipids in the butanol phase were analyzed by mass spectrometry to monitor the conversion of BODIPY-PC to BODIPY LCP by PLA_2_.

#### Liquid chromatography/mass spectrometry for targeted Phospholipase A2 activity analysis

The lipid samples were reconstituted in 100 µL of methanol ∶ water ∶ formic acid (74∶25∶1) containing 5 mM ammonium formate and analyzed with an Agilent 6400 QQQ (Agilent Technologies, Santa Clara, CA) coupled to an 1100 Series LC equipped with HPLC Chip interface (Agilent Technologies Santa Clara, CA). Solvent A consisted of methanol ∶ water ∶ formic acid (74 ∶ 25 ∶ 1) containing 5 mM ammonium formate and solvent B methanol ∶ formic acid (99 ∶ 1) containing 5 mM ammonium formate. The gradient was as follows: time 0 minutes, 40% B; time 5 minute, 70% B; time 7 minutes, 100% B; time 9 minutes, 100% B; time 9 minutes, 40% B; time 13 minutes, 40% B. The flow rate was 0.4 µL/min. Data were acquired for parent ions corresponding to the BODIPY-PC (986.9 m/z) and BODIPY LCP (320.2 m/z) in single ion monitoring (SIM) mode with the following source conditions: gas temperature 300°C, gas flow 4 L/minute, capillary voltage 1900 V. The fragmentation voltage was set to 200 V in all cases. Data were processed with Agilent Mass Hunter software version B03.01.

### Supplemental data

Supplemental data include three figure and three tables.

## Supporting Information

Figure S1 **PLA_2_ is activated in DENV-infected mosquito cells.** C6/36 cells were either mock-infected or infected with DENV at an MOI = 3. At the indicated time points, media was removed, and new media containing the fluorogenic phospholipase A substrate (BODIPY-PC) was added to the cells. The cells were incubated at 30°C for 30 min. Following the incubation, cells were washed and lipids were extracted and analyzed by mass spectrometry to monitor the conversion of BODIPY-PC to BODIPY LCP by PLA_2_. The graph represents the ratio of BODIPY-LPC/PC with time.
(TIF)

Figure S2 **Lipid homeostasis is altered in DENV infected mosquito cells.** A hierarchical clustering analysis of the results from the mass spectrometry analysis of lipid extracts from the 16K membrane fraction isolated at the 36 hr time point post treatment of C6/36 cells with the different conditions. The conditions included: uninfected cells (Mock), DENV-infected cells (DENV), UV-inactivated DENV treated cells (UV-DENV). Each condition included 3 replicates (denoted 1-3). Each horizontal row represents a differentially regulated metabolite. Each vertical row represents an individual sample. The samples from left to right include: DEN_1-3; cells infected with DENV, Mock_1-3; uninfected cells, UV-DEN_1-3; cells exposed to UV-inactivated DENV (UV-DENV). The Row Z-Score was calculated by subtracting the mean of the row from every value and then dividing the resulting values by the standard deviation of the row. The heatmap was created using the heatmap.2 function of the ‘gplots’ package in R [64].
(TIF)

Figure S3 **The FAS inhibitor, C75 alters Lipid homeostasis in DENV infected mosquito cells.** A hierarchical clustering analysis of the results from the mass spectrometry analysis of lipid extracts from the 16K membrane fraction isolated at the 36 hr time point post treatment of C6/36 cells with the different viruses and drug conditions. The conditions included: uninfected cells (Mock), uninfected cells treated with 25 µM C75 (C75 Mock), DENV-infected cells (DENV), DENV-infected cells treated with 25 µM C75 (C75 DENV), UV-inactivated DENV treated cells (UV-DENV). Each treatment included 3 replicates (denoted 1-3). Each horizontal row represents a differentially regulated metabolite. Each vertical row represents an individual sample. The Row Z-Score was calculated by subtracting the mean of the row from every value and then dividing the resulting values by the standard deviation of the row. The heatmap was created using the heatmap.2 function of the ‘gplots’ package in R [64].
(TIF)

Table S1 **Select list of lipid species from the whole cell analysis significantly regulated across conditions and time points (Anova p<0.05).** Conditions: Mock (uninfected cells), DENV (infectious dengue virus type 2, strain 16681), UV-DENV (UV-inactivated dengue virus type 2, strain 16681). Time points: 36 and 60 hr post-infection. A total of 7216 features were detected in the mass spectrometry analysis. Following Anova analysis, 677 features had a p<0.05. Structural identification was carried out on 100 lipids. For each condition and time point, the following information is provided: Treatment P; p-values from the Anova analysis on minimum observations data (p<0.1), Exact mass; mass of each lipid from http://www.lipidmaps.org, [M+H]^+^; Protonated molecular ion, NET; normalized elution time, total carbon/double bond; corresponds to each lipid species detected, LM_ID; identification for each lipid as displayed in LIPID MAPS, Formula, elemental composition of each lipid, PPM Error; difference in experimental mass compared to exact mass, Fold DENV/Mock; fold change of average abundance from 4 replicates of each treatment. Identity abbreviations were made for phoshatidylcholine (PC; O- fatty acid chain number means that an alkyl acyl linkage to the glycerol chain is present for the respective PC), phosphatidylethnolamine (PE), phosphatidylserine (PS), sphingomyelin (SM), ceramide (Cer), ceramide phosphoethanolamine (Cer-PE), lysophosphatidylcholine (LPC). The notation further indicates total number of carbons and double bonds however it does not discern redundancy associated with varying fatty acid composition for the same molecular weight. Accession numbers (LM_ID) were obtained from the Lipid Maps Gateway (lipidmaps.org).
(PDF)

Table S2 **Select list of lipid species from the analysis of the 16K membrane fraction (isolated from mosquito cells treated with conditions described below) regulated across conditions (p<0.05).** Conditions: Mock (uninfected cells), DENV (infectious dengue virus type 2, strain 16681), UV-DENV (UV-inactivated dengue virus type 2, strain 16681). A total of 484 features were detected in the mass spectrometry analysis following normalization of Mock, DENV and UV-DENV treated samples and 415 of these features were significantly regulated (p<0.05). Identification was successful for 68 lipids (ppm error <10). For each condition the following information is provided: pttest and pwilcox; p-values on minimum observation data (a measurement must be present in two of three replicates to be considered present), Exact mass; mass of each lipid from http://www.lipidmaps.org, M/Z; mass to charge ratio, RT; retention time, Formula, elemental composition of each lipid, PPM Error; difference in experimental mass compared to exact mass, Fold DENV/Mock; Fold UV-DENV/Mock; fold change of average abundance from 3 replicates of each treatment. Identity abbreviations were made for phoshatidylcholine (PC), phosphatidylethnolamine (PE), phosphatidylglycerol (PG, O- fatty acid chain number means that an alkyl acyl linkage to the glycerol chain is present for the respective PG), sphingomyelin (SM), monoacylglycerol (MG), diacylglycerol (DG), phosphatidic acid (PA). Accession numbers were obtained from the Lipid Maps Gateway (lipidmaps.org) and the Human Metabolome Database (hmdb.ca).
(PDF)

Table S3 **Select list of lipid species from the analysis of the 16K membrane fraction (isolated from mosquito cells treated with conditions described below) regulated across conditions (p<0.05).** Conditions: DENV (cells infected with DENV in the presence of vehicle only), DENV C75 (cells infected with DENV and treated with the FAS inhibitor C75). A total of 366 features were detected in the mass spectrometry analysis following normalization of DENV and DENV C75 treated samples and 314 of these features were significantly regulated (p<0.05). Identification was successful for 27 lipids (ppm error <10). For each condition the following information is provided: pttest and pwilcox; p-values on minimum observation data (a measurement must be present in two of three replicates to be considered present), Exact mass; mass of each lipid from http://www.lipidmaps.org, M/Z; mass to charge ratio, RT; retention time, Formula, elemental composition of each lipid, PPM Error; difference in experimental mass compared to exact mass, Fold DENV/DENV C75; fold change of average abundance from 3 replicates of each treatment. Identity abbreviations were made for phoshatidylinositol (PI), phosphatidyllglycerol (PG), monoacylglycerol (MG), diacylglycerol (DG), phosphatidic acid (PA). Accession numbers were obtained from the Lipid Maps Gateway (lipidmaps.org) and the Human Metabolome Database (hmdb.ca).
(PDF)

## References

[1] Visser B, Le Lann C, den Blanken FJ, Harvey JA, van Alphen JJ (2010). Loss of lipid synthesis as an evolutionary consequence of a parasitic lifestyle.. Proc Natl Acad Sci U S A.

[2] Diaz A, Wang X, Ahlquist P (2010). Membrane-shaping host reticulon proteins play crucial roles in viral RNA replication compartment formation and function.. Proc Natl Acad Sci U S A.

[3] Gillespie LK, Hoenen A, Morgan G, Mackenzie JM (2010). The endoplasmic reticulum provides the membrane platform for biogenesis of the flavivirus replication complex.. J Virol.

[4] Welsch S, Miller S, Romero-Brey I, Merz A, Bleck CK (2009). Composition and three-dimensional architecture of the dengue virus replication and assembly sites.. Cell Host Microbe.

[5] Jackson WT, Giddings TH, Taylor MP, Mulinyawe S, Rabinovitch M (2005). Subversion of cellular autophagosomal machinery by RNA viruses.. PLoS Biol.

[6] Lyle JM, Bullitt E, Bienz K, Kirkegaard K (2002). Visualization and functional analysis of RNA-dependent RNA polymerase lattices.. Science.

[7] Knoops K, Kikkert M, Worm SH, Zevenhoven-Dobbe JC, van der Meer Y (2008). SARS-coronavirus replication is supported by a reticulovesicular network of modified endoplasmic reticulum.. PLoS Biol.

[8] Munger J, Bennett BD, Parikh A, Feng XJ, McArdle J (2008). Systems-level metabolic flux profiling identifies fatty acid synthesis as a target for antiviral therapy.. Nat Biotechnol.

[9] Diamond DL, Syder AJ, Jacobs JM, Sorensen CM, Walters KA (2010). Temporal proteome and lipidome profiles reveal hepatitis C virus-associated reprogramming of hepatocellular metabolism and bioenergetics.. PLoS Pathog.

[10] Alvisi G, Madan V, Bartenschlager R (2011). Hepatitis C virus and host cell lipids: an intimate connection.. RNA Biol.

[11] Spencer CM, Schafer XL, Moorman NJ, Munger J (2011). Human cytomegalovirus induces the activity and expression of acetyl-coenzyme A carboxylase, a fatty acid biosynthetic enzyme whose inhibition attenuates viral replication.. J Virol.

[12] Wang X, Diaz A, Hao L, Gancarz B, den Boon JA (2011). Intersection of the multivesicular body pathway and lipid homeostasis in RNA replication by a positive-strand RNA virus.. J Virol.

[13] Morens DM, Fauci AS (2008). Dengue and hemorrhagic fever: a potential threat to public health in the United States.. JAMA.

[14] WHO report on global surveillance of epidemic –prone infectious disease – dengue and dengue haemorrhagic fever.. http://www.who.int/csr/resources/publications/dengue/CSR_ISR_2000_1/en/index.html.

[15] Zhang M, Zheng X, Wu Y, Gan M, He A (2010). Quantitative analysis of replication and tropisms of dengue virus type 2 in Aedes albopictus.. Am J Trop Med Hyg.

[16] Cao-Lormeau VM (2009). Dengue viruses binding proteins from *Aedes aegypti* and *Aedes polynesiensis* salivary glands.. Virol J.

[17] Tchankouo-Nguetcheu S, Khun H, Pincet L, Roux P, Bahut M (2010). Differential protein modulation in midguts of *Aedes aegypti* infected with chikungunya and dengue 2 viruses.. PLoS One.

[18] Hase T, Summers PL, Eckels KH, Baze WB (1987). An electron and immunoelectron microscopic study of dengue-2 virus infection of cultured mosquito cells: maturation events.. Arch Virol.

[19] Ko KK, Igarashi A, Fukai K (1979). Electron microscopic observations on *Aedes albopictus* cells infected with dengue viruses.. Arch Virol.

[20] Heaton NS, Perera R, Berger KL, Khadka S, Lacount DJ (2010). Dengue virus nonstructural protein 3 redistributes fatty acid synthase to sites of viral replication and increases cellular fatty acid synthesis.. Proc Natl Acad Sci U S A.

[21] Van Meer G, Voelker DR, Feigenson GW (2008). Membrane lipids: where they are and how they behave.. Nat Rev Mol Cell Biol.

[22] Jenkin HM, McMeans E, Anderson LE, Yang TK (1975). Comparison of phospholipid composition of *Aedes aegypti* and *Aedes albopictus* cells obtained from logarithmic and stationary phases of growth.. Lipids.

[23] Dennis RD, Wiegandt H (1993). Glycosphingolipids of the invertebrata as exemplified by a cestode *platyhelminth*, *Taenia crassiceps*, and a dipteran insect, *Calliphora vicina*.. Adv Lipid Res.

[24] Abeytunga DT, Glick JJ, Gibson NJ, Oland LA, Somogyi A (2004). Presence of unsaturated sphingomyelins and changes in their composition during the life cycle of the moth *Manduca sexta*.. J Lipid Res.

[25] Townsend D, Jenkin HM, Yang TK (1972). Lipid analysis of *Aedes aegypti* cells cultivated in vitro.. Biochim Biophys Acta.

[26] Butters TD, Hughes RC (1981). Phospholipids and Glycolipids in Subcellular Fractions of Mosquito Aedes aegypti Cells.. In Vitro.

[27] Cullis PR, de Kruijff B (1979). Lipid polymorphism and the functional roles of lipids in biological membranes.. Biochim Biophys Acta.

[28] Janmey PA, Kinnunen PK (2006). Biophysical properties of lipids and dynamic membranes.. Trends Cell Biol.

[29] Rivera R, Chun J (2008). Biological effects of lysophospholipids. Rev Physiol Biochem Pharmacol..

[30] Hishikawa D, Shindou H, Kobayashi S, Nakanishi H, Taguchi R (2008). Discovery of a lysophospholipid acyltransferase family essential for membrane asymmetry and diversity.. Proc Natl Acad Sci U S A.

[31] Allal C, Buisson-Brenac C, Marion V, Claudel-Renard C, Faraut T (2004). Human cytomegalovirus carries a cell-derived phospholipase A2 required for infectivity.. J Virol.

[32] Hannun YA, Obeid LM (2008). Principles of bioactive lipid signaling: lessons from sphingolipids.. Nat Rev Mol Cell Biol.

[33] Gault CR, Obeid LM, Hannun YA (2010). An overview of sphingolipid metabolism: from synthesis to breakdown.. Adv Exp Med Biol.

[34] Hannun YA, Obeid LM (2011). Many Ceramides.. J Biol Chem.

[35] Uchil PD, Satchidanandam V (2003). Architecture of the flaviviral replication complex. Protease, nuclease, and detergents reveal encasement within double-layered membrane compartments.. J Biol Chem.

[36] Ducharme NA, Bickel PE (2008). Minireview: Lipid Droplets in Lipogenesis and Lipolysis.. Endocrinol.

[37] Bligh EG, Dyer WJ (1959). A rapid method of total lipid extraction and purification.. Can J Biochem Physiol.

[38] Merrill AH, Sullards MC, Allegood JC, Kelly S, Wang E (2005). Sphingolipidomics: high-throughput, structure-specific, and quantitative analysis of sphingolipids by liquid chromatography tandem mass spectrometry.. Methods.

[39] Van Meer G (1989). Lipid traffic in animal cells.. Annu Rev Cell Biol.

[40] Martinez-Seara H, Róg T, Pasenkiewicz-Gierula M, Vattulainen L, Karttunen M (2008). Interplay of Unsaturated Phospholipids and Cholesterol in Membranes: Effect of the Double-Bond Position.. Biophys J.

[41] Roux A, Cuvelier D, Nassoy P, Prost J, Bassereau P (2005). Role of curvature and phase transition in lipid sorting and fission of membrane tubules.. EMBO J.

[42] Utermöhlen O, Herz J, Schramm M, Krönke M (2008). Fusogenicity of membranes: the impact of acid sphingomyelinase on innate immune responses.. Immunobiol.

[43] Chen WJ, Chen SL, Fang AH (1994). Phenotypic characteristics of dengue 2 virus persistently infected in a C6/36 clone of *Aedes albopictus* cells.. Intervirol.

[44] Smith WL, Merrill AH (2002). Sphingolipid metabolism and signaling minireview series.. J Biol Chem.

[45] Linkous A, Yazlovitskaya E (2010). Cytosolic phospholipase A2 as a mediator of disease pathogenesis.. Cell Microbiol.

[46] Zádori Z, Szelei J, Lacoste MC, Li Y, Gariépy S (2001). A viral phospholipase A2 is required for parvovirus infectivity.. Dev Cell.

[47] Chernomordik L (1996). Non-bilayer lipids and biological fusion intermediates.. Chem Phys Lipids.

[48] Davidsen J, Mouritsen OG, Jørgensen K (2002). Synergistic permeability enhancing effect of lysophospholipids and fatty acids on lipid membranes.. Biochim Biophys Acta.

[49] Lass A, Zimmermann R, Oberer M, Zechner R (2011). Lipolysis - a highly regulated multi-enzyme complex mediates the catabolism of cellular fat stores.. Prog Lipid Res.

[50] Zaitseva E, Yang ST, Melikov K, Pourmal S, Chernomordik LV (2010). Dengue virus ensures its fusion in late endosomes using compartment-specific lipids.. PLoS Pathog.

[51] Kuhajda FP, Pizer ES, Li JN, Mani NS, Frehywot GL (2000). Synthesis and antitumor activity of an inhibitor of fatty acid synthase. Proc.. Natl Acad Sci U S A.

[52] Jan JT, Chatterjee S, Griffin DE (2000). Sindbis virus entry into cells triggers apoptosis by activating sphingomyelinase, leading to the release of ceramide.. J Virol.

[53] Brojatsch J, Naughton J, Rolls MM, Zingler K, Young JAT (1996). CAR1, a TNFR-related protein, is a cellular receptor for cytopathic avian leukosis-sarcoma viruses and mediates apoptosis.. Cell.

[54] Hanon E, Meyer G, Vanderplasschen A, Dessy-Doize C, Thiry E (1998). Attachment but not penetration of bovine herpesvirus 1 is necessary to induce apoptosis in target cells.. J Virol.

[55] Ramsey-Ewing A, Moss B (1998). Apoptosis induced by a postbinding step of vaccinia virus entry into Chinese hamster ovary cells.. Virol.

[56] Kiebel GR, Auberry KJ, Jaitly N (2006). PRISM: a data management system for high-throughput proteomics.. Proteom.

[57] Monroe ME, Tolić N, Jaitly N, Shaw JL, Adkins JN (2007). VIPER: an advanced software package to support high-throughput LC-MS peptide identification.. Bioinform.

[58] Jaitly N, Monroe ME, Petyuk VA, Clauss TR, Adkins JN (2006). Robust algorithm for alignment of liquid chromatography-mass spectrometry analyses in an accurate mass and time tag data analysis pipeline.. Anal Chem.

[59] Ding J, Sorensen CM, Jaitly N (2008). Application of the accurate mass and time tag approach in studies of the human blood lipidome.. J Chromatogr B Analyt Technol Biomed Life Sci.

[60] Polpitiya AD, Qian WJ, Jaitly N (2008). DAnTE: a statistical tool for quantitative analysis of -omics data.. Bioinform.

[61] Callister SJ, Barry RC, Adkins JN (2006). Normalization approaches for removing systematic biases associated with mass spectrometry and label-free proteomics.. J Proteome Res.

[62] Trygg J, Holmes E, Lundstedt T (2007). Chemometrics in metabonomics.. J Proteome Res.

[63] Riley CP, Gough ES, He J, Jandhyala SS, Kennedy B (2007). The Proteome Discovery Pipeline - A Data Analysis Pipeline for Mass Spectrometry-Based Differential Proteomics Discovery.. Open Proteomics J.

[64] R Development Core Team (2007). R: A language and environment for statistical computing.. http://www.R-project.org.

[65] Merrill AH, Sullards MC, Allegood JC, Kelly S (2005). Sphingolipidomics: high-throughput, structure-specific, and quantitative analysis of sphingolipids by liquid chromatography tandem mass spectrometry.. Methods.

