# Supplementary figures and images for: Dengue Virus Infection Perturbs Lipid Homeostasis in Infected Mosquito Cells
Source: PLoS Pathog. 2012 Mar 22;8(3):e1002584. doi: 10.1371/journal.ppat.1002584 (PMC3310792; doi:10.1371/journal.ppat.1002584)

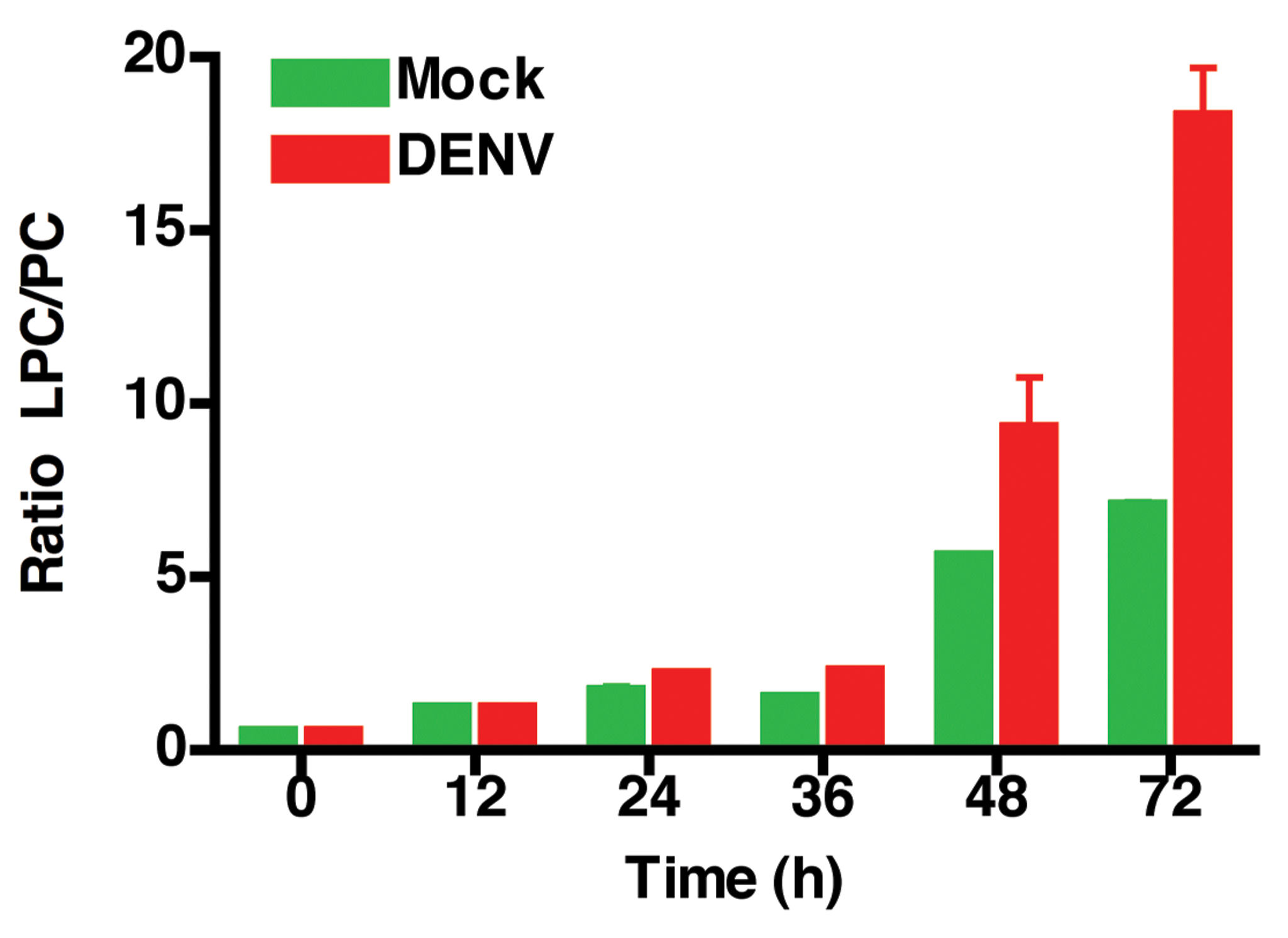

Supplement: Figure S1 — PLA2 is activated in DENV-infected mosquito cells. C6/36 cells were either mock-infected or infected with DENV at an MOI = 3. At the indicated time points, media was removed, and new media containing the fluorogenic phospholipase A substrate (BODIPY-PC) was added to the cells. The cells were incubated at 30°C for 30 min. Following the incubation, cells were washed and lipids were extracted and analyzed by mass spectrometry to monitor the conversion of BODIPY-PC to BODIPY LCP by PLA2. The graph represents the ratio of BODIPY-LPC/PC with time. (TIF) [file ppat.1002584.s001.tif]

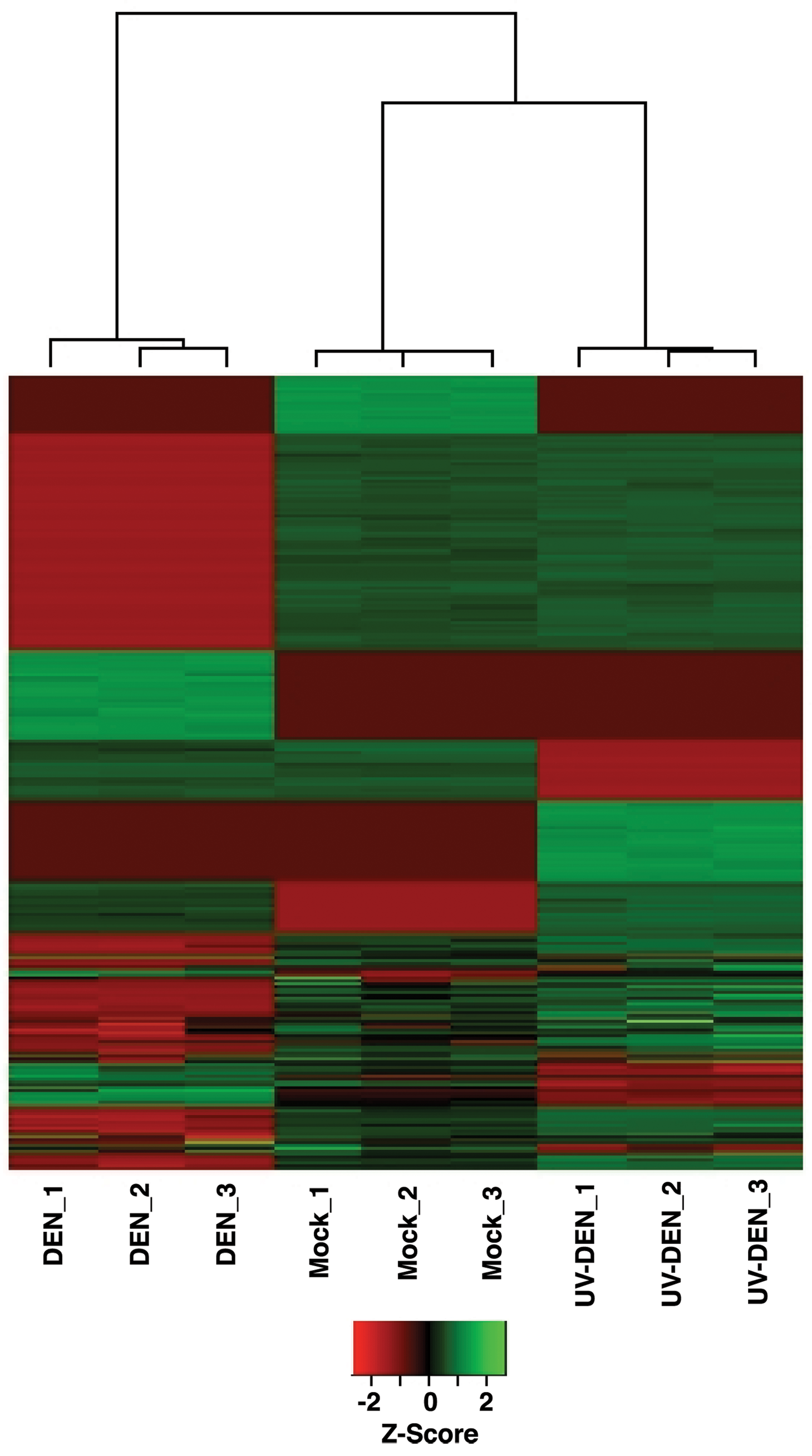

Supplement: Figure S2 — Lipid homeostasis is altered in DENV infected mosquito cells. A hierarchical clustering analysis of the results from the mass spectrometry analysis of lipid extracts from the 16K membrane fraction isolated at the 36 hr time point post treatment of C6/36 cells with the different conditions. The conditions included: uninfected cells (Mock), DENV-infected cells (DENV), UV-inactivated DENV treated cells (UV-DENV). Each condition included 3 replicates (denoted 1-3). Each horizontal row represents a differentially regulated metabolite. Each vertical row represents an individual sample. The samples from left to right include: DEN_1-3; cells infected with DENV, Mock_1-3; uninfected cells, UV-DEN_1-3; cells exposed to UV-inactivated DENV (UV-DENV). The Row Z-Score was calculated by subtracting the mean of the row from every value and then dividing the resulting values by the standard deviation of the row. The heatmap was created using the heatmap.2 function of the ‘gplots’ package in R [64]. (TIF) [file ppat.1002584.s002.tif]

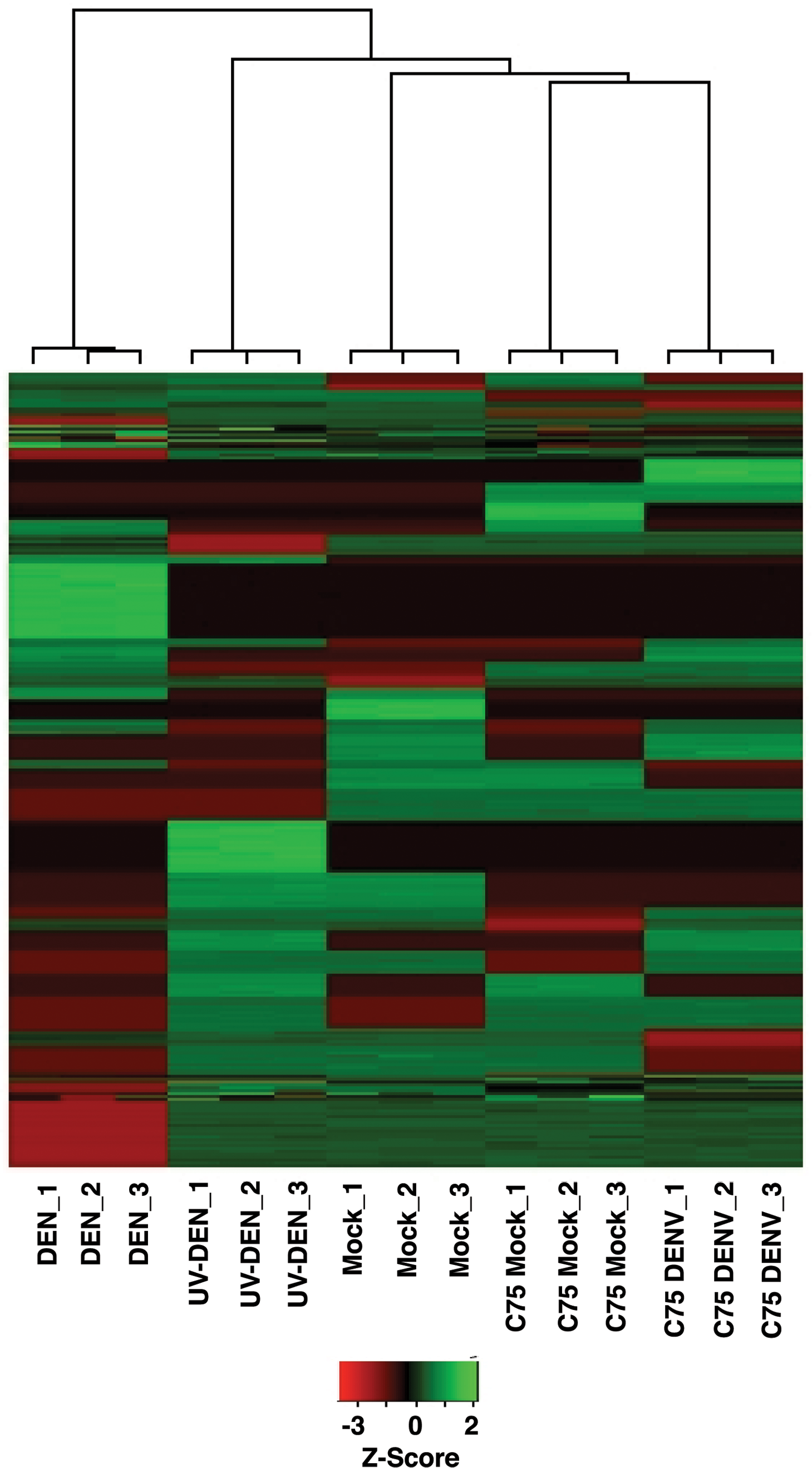

Supplement: Figure S3 — The FAS inhibitor, C75 alters Lipid homeostasis in DENV infected mosquito cells. A hierarchical clustering analysis of the results from the mass spectrometry analysis of lipid extracts from the 16K membrane fraction isolated at the 36 hr time point post treatment of C6/36 cells with the different viruses and drug conditions. The conditions included: uninfected cells (Mock), uninfected cells treated with 25 µM C75 (C75 Mock), DENV-infected cells (DENV), DENV-infected cells treated with 25 µM C75 (C75 DENV), UV-inactivated DENV treated cells (UV-DENV). Each treatment included 3 replicates (denoted 1-3). Each horizontal row represents a differentially regulated metabolite. Each vertical row represents an individual sample. The Row Z-Score was calculated by subtracting the mean of the row from every value and then dividing the resulting values by the standard deviation of the row. The heatmap was created using the heatmap.2 function of the ‘gplots’ package in R [64]. (TIF) [file ppat.1002584.s003.tif]
