# Supplementary material for: Dengue Virus Infection Perturbs Lipid Homeostasis in Infected Mosquito Cells
Source: PLoS Pathog. 2012 Mar 22;8(3):e1002584. doi: 10.1371/journal.ppat.1002584 (PMC3310792; doi:10.1371/journal.ppat.1002584)
Supplement: Table S1 — Select list of lipid species from the whole cell analysis significantly regulated across conditions and time points (Anova p<0.05). Conditions: Mock (uninfected cells), DENV (infectious dengue virus type 2, strain 16681), UV-DENV (UV-inactivated dengue virus type 2, strain 16681). Time points: 36 and 60 hr post-infection. A total of 7216 features were detected in the mass spectrometry analysis. Following Anova analysis, 677 features had a p<0.05. Structural identification was carried out on 100 lipids. For each condition and time point, the following information is provided: Treatment P; p-values from the Anova analysis on minimum observations data (p<0.1), Exact mass; mass of each lipid from http://www.lipidmaps.org, [M+H]+; Protonated molecular ion, NET; normalized elution time, total carbon/double bond; corresponds to each lipid species detected, LM_ID; identification for each lipid as displayed in LIPID MAPS, Formula, elemental composition of each lipid, PPM Error; difference in experimental mass compared to exact mass, Fold DENV/Mock; fold change of average abundance from 4 replicates of each treatment. Identity abbreviations were made for phoshatidylcholine (PC; O- fatty acid chain number means that an alkyl acyl linkage to the glycerol chain is present for the respective PC), phosphatidylethnolamine (PE), phosphatidylserine (PS), sphingomyelin (SM), ceramide (Cer), ceramide phosphoethanolamine (Cer-PE), lysophosphatidylcholine (LPC). The notation further indicates total number of carbons and double bonds however it does not discern redundancy associated with varying fatty acid composition for the same molecular weight. Accession numbers (LM_ID) were obtained from the Lipid Maps Gateway (lipidmaps.org). (PDF) [file ppat.1002584.s004.pdf]

Supplementary Table 1

Treatment (P)	Exact Mass	[M+H]+	NET	Total Carbon/Dl	LM_ID	FORMULA	PPM Error	Fold Den /Mock
**Mock vs Den 36hr**								
5.70E-04	493.319547	494.327367	0.202519899	LPC 16:1	LMGP01050021	C24H48NO7P	5.568429861	2.84633556
4.70E-04	495.3351819	496.3430019	0.225881771	LPC 16:0	LMGP01050018	C24H50NO7P	5.515285744	5.70916973
1.42E-02	507.3713078	508.3791278	0.259450505	LPC p-18:0 or O-	LMGP01070009	C26H54NO6P	4.863917146	3.075249133
1.27E-03	521.3509477	522.3587677	0.222334181	LPC 18:1	LMGP01050030	C26H52NO7P	5.462185438	2.915070791
1.25E-02	689.5401977	690.5480177	0.397908715	PC O-30:1	LMGP01020014	C38H76NO7P	6.232742922	2.081571725
2.44E-03	717.5716173	718.5794373	0.398614135	PC O-32:1	LMGP01020016	C40H80NO7P	6.155939123	1.984340482
4.15E-02	719.5876173	720.5954373	0.486556448	PC O-32:0	LMGP01020015	C40H82NO7P	6.625088411	1.658492354
5.19E-03	745.6029093	746.6107293	0.453108572	PC (16:0e/18:1;1	LMGP01020003	C42H84NO7P	5.913772627	1.783402315
2.91E-02	747.619111	748.626931	0.575690701	PC O-34:0	LMGP01020086	C42H86NO7P	6.635775955	4.303059026
1.16E-02	773.6346917	774.6425117	0.524861954	PC (16:0p/20:0;1	LMGP01020052	C44H88NO7P	6.32305012	2.092810706
1.45E-02	779.5511791	780.5589991	0.321648936	PC 36:5	LMGP01011305	C44H78NO8P	6.053648169	1.944815964
7.47E-03	781.5666768	782.5744968	0.355787216	PC 36:4	LMGP01011056	C44H80NO8P	5.843169649	1.890537453
0.01770563	787.6138126	788.6216326	0.477367579	PC 36:1	LMGP01010761	C44H86NO8P	6.034211948	1.889083063
9.52E-03	803.5515391	804.5593591	0.30519687	PC 38:7	LMGP01010696	C46H78NO8P	6.320854179	1.872550202
6.41E-02	805.5671035	806.5749235	0.339317721	PC 38:6	LMGP01010649	C46H80NO8P	6.19877715	2.029369367
5.68E-03	807.5827718	808.5905918	0.350068809	PC 38:5	LMGP01010644	C46H82NO8P	6.20596585	1.842116429
4.68E-02	815.6453964	816.6532164	0.557540948	PC 38:1	LMGP01010786	C46H90NO8P	6.174780267	1.702561539
2.78E-02	453.288309	454.296129	0.225708132	LPE 16:0	LMGP02050002	C21H44NO7P	6.196977402	2.335860578
9.27E-03	479.3038817	480.3117017	0.224633514	LPE 18:1	LMGP02050004	C23H46NO7P	5.699339549	2.227590249
1.47E-02	663.4881255	664.4959455	0.383211376	PE 30:0	LMGP02010302	C35H70NO8P	6.428943125	0.830705785
2.45E-02	691.5195301	692.5273501	0.444906928	PE 32:0	LMGP02010017	C37H74NO8P	6.319601149	0.700600412
4.58E-02	715.5199495	716.5277695	0.364265384	PE 34:2	LMGP02010042	C39H74NO8P	6.693778508	1.425745712
6.85E-02	745.5668685	746.5746885	0.482835154	PE 36:1	LMGP02010026	C41H80NO8P	6.382432713	1.469935504
4.13E-02	717.5356843	718.5435043	0.42173318	PE 34:1	LMGP02010009	C39H76NO8P	6.793157774	1.241120276
2.61E-02	773.5985924	774.6064124	0.564948553	PE 38:1	LMGP02010135	C43H84NO8P	6.699126354	1.720397124

1.32E-02	704.5880253	705.5958453	0.41755266	SM(d18:0/16:0)	LMSP03010004	C39H81N2O6P	6.869735332	1.826860316
6.76E-04	730.6036905	731.6115105	0.436458594	SM(d18:1/18:0)	LMSP03010001	C41H83N2O6P	6.645918071	1.59231592
6.76E-04	730.6036905	731.6115105	0.436458594	SM(d18:0/18:1(	LMSP03010031	C41H83N2O6P	6.645918071	1.59231592
2.83E-02	758.6351933	759.6430133	0.516324213	SM(d18:1/20:0)	LMSP03010005	C43H87N2O6P	6.667676074	1.575489046
2.73E-02	786.6668188	787.6746388	0.647287175	SM(d18:1/22:0)	LMSP03010006	C45H91N2O6P	6.843859074	2.223605831
1.49E-02	812.6824056	813.6902256	0.596711373	SM(d18:1/24:1(	LMSP03010007	C47H93N2O6P	6.547003845	1.804907249
3.98E-02	814.6988949	815.7067149	0.786144633	SM(d18:1/24:0)	LMSP03010008	C47H95N2O6P	7.561010103	2.964080988
3.98E-02	814.6988949	815.7067149	0.786144633	SM(d18:0/24:1(	LMSP03010023	C47H95N2O6P	7.561010103	2.964080988
2.03E-02	539.5305375	540.5383575	0.405250914	Cer(d18:0/16:0)	LMSP02020001	C34H69NO3	5.212892442	2.992163729
1.90E-02	565.546695	566.554515	0.436733191	Cer(d18:1/18:0)	LMSP02010006	C36H71NO3	5.870460422	1.545681792
1.24E-02	593.578296	594.586116	0.509251945	Cer(d18:1/20:0)	LMSP02010007	C38H75NO3	6.100327646	1.976746158
1.86E-02	621.6099393	622.6177593	0.627423975	Cer(d18:1/22:0)	LMSP02010008	C40H79NO3	6.3775127	1.880107083
0.010297163	647.6259132	648.6337332	0.584752798	Cer(d18:1/24:1(	LMSP02010009	C42H81NO3	6.621458942	2.220459657
5.91E-03	658.5091829	659.5170029	0.365175089	CER-PE d18:1/16:1			6.428045833	0.630084333
3.56E-02	686.5409151	687.5487351	0.413428042	CER-PE d18:1/18:1			6.795125531	0.754138565

**Mock vs UV-DENV 36hr**								
4.92E-02	689.5401977	690.5480177	0.397908715	PC O-30:1	LMGP01020014	C38H76NO7P	6.232742922	1.080169664
1.05E-02	691.5560233	692.5638433	0.411461787	PC O-30:0	LMGP01020012	C38H78NO7P	6.468498263	1.3970845
1.03E-02	703.5194574	704.5272774	0.330359699	PC 30:1	LMGP01010485	C38H74NO8P	6.108468224	1.102093358
5.23E-03	715.5492994	716.5571194	0.237883988	PC O-32:2 or P-3	LMGP01020017	C40H78NO7P	3.145266054	0.971196157
1.55E-02	729.5355247	730.5433447	0.324204633	PC 32:2	LMGP01010494	C40H76NO8P	6.462646862	1.171126621
5.66E-02	743.5868644	744.5946844	0.436414521	PC O-34:2 or p-3	LMGP01020039	C42H82NO7P	5.398725912	1.062094499
6.42E-02	757.5668252	758.5746452	0.360999159	PC 34:2	LMGP01010585	C42H80NO8P	6.224176127	1.133395569
2.32E-02	777.535513	778.543333	0.2984784	PC 36:6	LMGP01010953	C44H76NO8P	6.048634909	1.159034312
3.97E-02	781.5666768	782.5744968	0.355787216	PC 36:4	LMGP01011056	C44H80NO8P	5.843169649	1.214038621
9.00E-03	783.5825143	784.5903343	0.363478216	PC 36:3	LMGP01010896	C44H82NO8P	6.067425905	1.137889404
3.53E-02	785.5982148	786.6060348	0.404637242	PC 36:2	LMGP01010966	C44H84NO8P	6.116140918	1.069683304

2.67E-02	789.6299754	790.6377954	0.6093064	PC 36:0	LMGP01010006	C44H88NO8P	6.668231038	1.568816859
1.29E-02	801.6664401	802.6742601	0.628307212	PC O-38:1	LMGP01020099	C46H92NO7P	6.661293656	1.727539527
9.08E-03	803.5515391	804.5593591	0.30519687	PC 38:7	LMGP01010696	C46H78NO8P	6.320854179	1.121240577
1.57E-02	817.7218972	818.7297172	0.813940313	PC O2-40:0	LMGP01040072	C48H100NO6P	8.429200591	1.065196628
1.75E-02	853.5735849	854.5814049	0.372140982	PC 42:10	LMGP01011058	C50H80NO8P	13.44354425	0.946628564
5.46E-03	607.4250596	608.4328796	0.303505944	PE 26:0	LMGP02010058	C31H62NO8P	6.255296366	0.860069833
5.65E-02	635.4567044	636.4645244	0.340256167	PE 28:0	LMGP02010016	C33H66NO8P	6.521966014	0.862246615
4.44E-02	687.4886148	688.4964348	0.328023921	PE 32:2	LMGP02010019	C37H70NO8P	6.916235095	1.163696444
4.41E-02	703.5558784	704.5636984	0.46124096	PE O-34:1	LMGP02020018	C39H78NO7P	6.152214433	1.398798481
2.08E-02	713.5037658	714.5115858	0.331687984	PE 34:3	LMGP02010041	C39H72NO8P	5.964685246	1.297290184
4.45E-04	719.5510601	720.5588801	0.388924075	PE 34:0	LMGP02010024	C39H78NO8P	6.39305487	0.847787849
2.12E-03	735.4878162	736.4956362	0.299669066	PE 36:6	LMGP02010029	C41H70NO8P	5.37904394	1.241323262
1.37E-03	739.5199057	740.5277257	0.358025563	PE 36:4	LMGP02010012	C41H74NO8P	6.417312662	1.062074505
2.26E-04	747.5824683	748.5902883	0.445956792	PE 36:0	LMGP02010097	C41H82NO8P	6.298073929	1.059968845
1.00E-02	767.5511584	768.5589784	0.401985333	PE 38:4	LMGP02010027	C43H78NO8P	6.121323262	1.032409809
3.58E-03	785.5257681	786.5335881	0.305682618	PS 36:3	LMGP03010018	C42H76NO10P	6.541012169	0.859117154
1.26E-03	702.5721519	703.5799719	0.399650504	SM(d18:1/16:0)	LMSP03010003	C39H79N2O6P	6.571467895	1.068805993
5.92E-02	728.5879586	729.5957786	0.412634798	SM(d18:1/18:1(	LMSP03010029	C41H81N2O6P	6.551894277	1.263406718
8.60E-02	730.6036905	731.6115105	0.436458594	SM(d18:1/18:0)	LMSP03010001	C41H83N2O6P	6.645918071	1.072660429
8.60E-02	730.6036905	731.6115105	0.436458594	SM(d18:0/18:1(	LMSP03010031	C41H83N2O6P	6.645918071	1.072660429
5.33E-02	814.6988949	815.7067149	0.786144633	SM(d18:1/24:0)	LMSP03010008	C47H95N2O6P	7.561010103	1.300071909
5.33E-02	814.6988949	815.7067149	0.786144633	SM(d18:0/24:1(	LMSP03010023	C47H95N2O6P	7.561010103	1.300071909
4.33E-02	632.4933509	633.5011709	0.343500327	CER-PE (d18:1/14:0)			6.404693328	2.009538877

**Mock vs Den 60hr**								
9.61E-02	493.319547	494.327367	0.202519899	LPC 16:1	LMGP01050021	C24H48NO7P	5.568429861	1.463380564
2.07E-02	495.3351819	496.3430019	0.225881771	LPC 16:0	LMGP01050018	C24H50NO7P	5.515285744	1.774327424
1.12E-02	507.3713078	508.3791278	0.259450505	LPC p-18:0 or O-	LMGP01070009	C26H54NO6P	4.863917146	1.908240913
6.71E-02	689.5401977	690.5480177	0.397908715	PC O-30:1	LMGP01020014	C38H76NO7P	6.232742922	1.58485063

4.42E-02	705.5350823	706.5429023	0.379901525	PC 30:0	LMGP01010438	C38H76NO8P	6.055440726	0.682470041
2.08E-02	717.5716173	718.5794373	0.398614135	PC O-32:1	LMGP01020016	C40H80NO7P	6.155939123	1.492060267
2.14E-02	739.5413238	740.5491438	0.392680143	PC O-34:4	LMGP01020021	C42H78NO7P	13.82756888	0.341994588
7.02E-02	743.5868644	744.5946844	0.436414521	PC O-34:2 or p-3	LMGP01020039	C42H82NO7P	5.398725912	1.583762111
4.59E-02	745.6029093	746.6107293	0.453108572	PC (16:0e/18:1;1	LMGP01020003	C42H84NO7P	5.913772627	1.550191793
7.13E-02	765.5565897	766.5644097	0.375664212	PC O-36:5 or p-3	LMGP01020058	C44H80NO7P	13.85939732	0.374609824
9.38E-02	769.5663687	770.5741887	0.345663763	PC 35:3	LMGP01010611	C43H80NO8P	5.5339263	3.194431883
1.33E-02	771.618765	772.626585	0.437848068	PC O-36:2 or p-3	LMGP01030013	C44H86NO7P	5.980968597	1.942078446
1.49E-02	773.6346917	774.6425117	0.524861954	PC (16:0p/20:0;1	LMGP01020052	C44H88NO7P	6.32305012	1.575430327
5.30E-02	785.5982148	786.6060348	0.404637242	PC 36:2	LMGP01010966	C44H84NO8P	6.116140918	0.7202355
0.034365927	795.6119863	796.6198063	0.284435598	PC O-38:4	LMGP01020062	C46H86NO7P	2.719534337	3.228350963
1.69E-02	801.6664401	802.6742601	0.628307212	PC O-38:1	LMGP01020099	C46H92NO7P	6.661293656	2.102668773
0.035802206	803.5515391	804.5593591	0.30519687	PC 38:7	LMGP01010696	C46H78NO8P	6.320854179	1.933601926
7.09E-02	851.5575277	852.5653477	0.32637625	PC 42:11	LMGP01011059	C50H78NO8P	12.99717692	1.500993745
4.99E-02	853.5735849	854.5814049	0.372140982	PC 42:10	LMGP01011058	C50H80NO8P	13.44354425	1.786501809
2.87E-02	481.3194539	482.3272739	0.247755053	LPE 18:0	LMGP02050001	C23H48NO7P	5.513832054	0.521291677
1.06E-02	663.4881255	664.4959455	0.383211376	PE 30:0	LMGP02010302	C35H70NO8P	6.428943125	0.542125534
4.91E-02	691.5195301	692.5273501	0.444906928	PE 32:0	LMGP02010017	C37H74NO8P	6.319601149	0.622835939
5.20E-02	767.5511584	768.5589784	0.401985333	PE 38:4	LMGP02010027	C43H78NO8P	6.121323262	0.576192214
9.73E-02	785.5257681	786.5335881	0.305682618	PS 36:3	LMGP03010018	C42H76NO10P	6.541012169	0.527461432
4.41E-03	732.6194417	733.6272617	0.483677031	SM(d18:0/18:0)	LMSP03010020	C41H85N2O6P	6.765768493	2.746166783
8.88E-03	786.6668188	787.6746388	0.647287175	SM(d18:1/22:0)	LMSP03010006	C45H91N2O6P	6.843859074	1.6747704
7.40E-02	812.6824056	813.6902256	0.596711373	SM(d18:1/24:1(	LMSP03010007	C47H93N2O6P	6.547003845	1.518229184
6.13E-02	539.5305375	540.5383575	0.405250914	Cer(d18:0/16:0)	LMSP02020001	C34H69NO3	5.212892442	2.078336797
8.57E-03	567.5621921	568.5700121	0.464764162	Cer(d18:0/18:0)	LMSP02020008	C36H73NO3	5.58021256	2.400963331
4.50E-03	595.5938251	596.6016451	0.549369722	Cer(d18:0/20:0)	LMSP02020009	C38H77NO3	5.876690492	2.876748293

**Mock vs UV-Den 60hr**								
2.22E-02	495.3351819	496.3430019	0.225881771	LPC 16:0	LMGP01050018	C24H50NO7P	5.515285744	0.964100363

1.46E-03	507.3713078	508.3791278	0.259450505	LPC p-18:0 or O-	LMGP01070009	C26H54NO6P	4.863917146	2.232692095
9.74E-02	691.5560233	692.5638433	0.411461787	PC O-30:0	LMGP01020012	C38H78NO7P	6.468498263	0.841738911
8.83E-02	705.5350823	706.5429023	0.379901525	PC 30:0	LMGP01010438	C38H76NO8P	6.055440726	0.825708518
1.09E-02	717.5716173	718.5794373	0.398614135	PC O-32:1	LMGP01020016	C40H80NO7P	6.155939123	1.452384463
6.56E-02	719.5876173	720.5954373	0.486556448	PC O-32:0	LMGP01020015	C40H82NO7P	6.625088411	1.482570491
5.18E-02	739.5413238	740.5491438	0.392680143	PC O-34:4	LMGP01020021	C42H78NO7P	13.82756888	0.680247391
7.11E-02	745.6029093	746.6107293	0.453108572	PC (16:0e/18:1;1	LMGP01020003	C42H84NO7P	5.913772627	1.560881376
8.06E-02	759.5760449	760.5838649	0.414978015	PC 34:1	LMGP01010005	C42H82NO8P	2.257965004	1.01916648
4.95E-02	773.6346917	774.6425117	0.524861954	PC (16:0p/20:0;1	LMGP01020052	C44H88NO7P	6.32305012	1.391308049
0.043896138	813.6297323	814.6375523	0.462990088	PC 38:2	LMGP01010640	C46H88NO8P	6.172747629	1.887402496
2.31E-02	815.6453964	816.6532164	0.557540948	PC 38:1	LMGP01010786	C46H90NO8P	6.174780267	1.40550688
8.83E-02	877.5737285	878.5815485	0.340678093	PC 44:12	LMGP01011119	C52H80NO8P	13.23951874	0.662496092
9.51E-02	425.2569868	426.2648068	0.205633213	LPE 14:0	LMGP02050003	C19H40NO7P	6.553256852	0.849512707
4.18E-03	453.288309	454.296129	0.225708132	LPE 16:0	LMGP02050002	C21H44NO7P	6.196977402	0.836609199
1.63E-03	481.3194539	482.3272739	0.247755053	LPE 18:0	LMGP02050001	C23H48NO7P	5.513832054	0.637258779
7.28E-02	579.3935371	580.4013571	0.275257901	PE 24:0	LMGP02010015	C29H58NO8P	6.173907466	0.727753316
2.68E-02	607.4250596	608.4328796	0.303505944	PE 26:0	LMGP02010058	C31H62NO8P	6.255296366	0.641856227
3.43E-02	635.4567044	636.4645244	0.340256167	PE 28:0	LMGP02010016	C33H66NO8P	6.521966014	0.711682714
0.018784442	663.4881255	664.4959455	0.383211376	PE 30:0	LMGP02010302	C35H70NO8P	6.428943125	0.650405652
9.19E-04	691.5195301	692.5273501	0.444906928	PE 32:0	LMGP02010017	C37H74NO8P	6.319601149	0.667277452
7.68E-02	717.5356843	718.5435043	0.42173318	PE 34:1	LMGP02010009	C39H76NO8P	6.793157774	0.995917799
1.75E-02	739.5199057	740.5277257	0.358025563	PE 36:4	LMGP02010012	C41H74NO8P	6.417312662	0.777587508
6.02E-02	745.5668685	746.5746885	0.482835154	PE 36:1	LMGP02010026	C41H80NO8P	6.382432713	0.950669697
6.71E-02	763.51965	764.52747	0.328266645	PE 38:6	LMGP02010095	C43H74NO8P	5.880695283	0.655396547
8.36E-03	767.5511584	768.5589784	0.401985333	PE 38:4	LMGP02010027	C43H78NO8P	6.121323262	0.614331239
4.61E-02	787.5414408	788.5492608	0.411520119	PS 36:2	LMGP03010013	C42H78NO10P	6.553094925	0.673700855
0.025410831	704.5880253	705.5958453	0.41755266	SM(d18:0/16:0)	LMSP03010004	C39H81N2O6P	6.869735332	0.756099322
0.07807743	539.5305375	540.5383575	0.405250914	Cer(d18:0/16:0)	LMSP02020001	C34H69NO3	5.212892442	0.628969109
9.74E-03	621.6099393	622.6177593	0.627423975	Cer(d18:1/22:0)	LMSP02010008	C40H79NO3	6.3775127	1.991728417

5.32E-02	647.6259132	648.6337332	0.584752798	Cer(d18:1/24:1(	LMSP02010009	C42H81NO3	6.621458942	1.789645524
5.31E-03	649.6413634	650.6491834	0.760703851	Cer(d18:1/24:0)	LMSP02010012	C42H83NO3	6.29335809	2.000220433
3.40E-02	688.5563818	689.5642018	0.437363244	CER-PE d18:1/18:0			6.509022777	0.948604845
3.40E-02	688.5563818	689.5642018	0.437363244	Etn-1-P-Cer(d14	LMSP03020001	C38H77N2O6P	6.530807769	0.948604845
