# Supplementary material for: Dengue Virus Infection Perturbs Lipid Homeostasis in Infected Mosquito Cells
Source: PLoS Pathog. 2012 Mar 22;8(3):e1002584. doi: 10.1371/journal.ppat.1002584 (PMC3310792; doi:10.1371/journal.ppat.1002584)
Supplement: Table S2 — Select list of lipid species from the analysis of the 16K membrane fraction (isolated from mosquito cells treated with conditions described below) regulated across conditions (p<0.05). Conditions: Mock (uninfected cells), DENV (infectious dengue virus type 2, strain 16681), UV-DENV (UV-inactivated dengue virus type 2, strain 16681). A total of 484 features were detected in the mass spectrometry analysis following normalization of Mock, DENV and UV-DENV treated samples and 415 of these features were significantly regulated (p<0.05). Identification was successful for 68 lipids (ppm error <10). For each condition the following information is provided: pttest and pwilcox; p-values on minimum observation data (a measurement must be present in two of three replicates to be considered present), Exact mass; mass of each lipid from http://www.lipidmaps.org, M/Z; mass to charge ratio, RT; retention time, Formula, elemental composition of each lipid, PPM Error; difference in experimental mass compared to exact mass, Fold DENV/Mock; Fold UV-DENV/Mock; fold change of average abundance from 3 replicates of each treatment. Identity abbreviations were made for phoshatidylcholine (PC), phosphatidylethnolamine (PE), phosphatidylglycerol (PG, O- fatty acid chain number means that an alkyl acyl linkage to the glycerol chain is present for the respective PG), sphingomyelin (SM), monoacylglycerol (MG), diacylglycerol (DG), phosphatidic acid (PA). Accession numbers were obtained from the Lipid Maps Gateway (lipidmaps.org) and the Human Metabolome Database (hmdb.ca). (PDF) [file ppat.1002584.s005.pdf]

p.ttest	p.wilcox	Exact Mass	M/Z	RT	ID (Total carbon/double bond)	Formula	PPM Error	Fold DENV/Mock
0.0119212	0.0119212	884.54	883.532532	20.745	PI (38:5)	C47H81O13P	0.53361069	0.287375
0.01092014	0.01092014	884.54	883.532532	20.745	PI (38:5)	C47H81O13P	0.53361069	
0.00858658	0.2	836.54	835.532593	21.0543	PI (34:1)	C43H81O13P	0.637148254	3.27101
0.01196713	0.01196713	836.54	835.532593	21.0543	PI (34:1)	C43H81O13P	0.637148254	
0.01059142	0.01059142	856.51	855.500183	19.8827	PI (36:5)	C45H77O13P	-2.191451355	0.1
0.01080327	0.01080327	786.58	785.579041	28.5617	PG (O-38:3)	C44H83O9P	8.875130311	0.127265
0.00957995	0.00957995	437.290619	436.28244	23.5048	PE(P-16:0e/0:0)	C21H44NO6P	-0.546547284	10.51758
0.00839554	0.00839554	437.290619	436.282623	23.5593	PE(P-16:0e/0:0)	C21H44NO6P	-0.546547284	
0.01339142	0.2	717.53	716.521912	22.1253	PC (31:1)	C39H76NO8P	-0.206263153	1.970255
0.01721421	0.1	717.53	716.521912	22.1253	PC (31:1)	C39H76NO8P	-0.206263153	
0.01212817	0.01212817	745.562134	744.554199	22.9715	PC (33:1)	C41H80NO8P	0.006706349	0.1
0.01360544	0.1	745.562134	744.554199	22.9715	PC (33:1)	C41H80NO8P	0.006706349	
0.00954852	0.00954852	661.468262	660.4599	19.7488	PE (30:1)	C35H68NO8P		Not significant
0.0024705	0.2	689.499573	688.490845	21.111	PC (29:1)	C37H72NO8P	-1.142857851	Not significant
0.0107848	0.0107848	256.240234	255.232941	8.59932	Palmitic acid	C16H32O2	2.524974279	10.384106
0.0096012	0.0096012	256.240234	255.232605	8.57512	Palmitic acid	C16H32O2	2.524974279	
0.01086703	0.01086703	282.25589	281.24823	8.9167	Oleic acid	C18H34O2	0.992007642	0.145958
0.03288563	0.2	282.25589	281.24823	8.9167	Oleic acid	C18H34O2	0.992007642	
0.01046164	0.01046164	284.271515	283.263794	11.4335	Stearic acid	C18H36O2	0.770390238	2.4
0.00880594	0.00880594	284.271515	283.264069	11.0419	Stearic acid	C18H36O2	0.770390238	
0.00880971	0.00880971	358.31	357.300385	32.1102	MG (18:0)[rac]	C21H42O4	-4.674723005	10.415623
0.00903598	0.00903598	358.308319	357.300598	30.5262	MG (18:0)	C21H42O4	0.611205457	10.299662
0.01165203	0.1	358.308319	357.300598	22.7758	MG (18:0)	C21H42O4	0.611205457	
0.01097097	0.1	330.277008	329.269653	20.9483	MG (16:0)	C19H38O4	1.771240461	1.665827
0.01060442	0.01060442	330.277008	329.269257	21.0968	MG (16:0)	C19H38O4	1.771240461	
0.01040695	0.01040695	625.57	624.556091	28.912	DG (36:2)	C39H67D5O5	-9.541697972	10.290444
0.01039105	0.01039105	606.43	605.426941	22.8297	PA (29:0)	C32H63O8P	8.04874429	9.613046
0.01554847	0.1	537.512085	536.504822	27.7823	N-Palmitoylsphingosine	C34H67NO3	1.259506565	2.751471
0.00041734	0.1	537.512085	536.503784	27.6613	N-Palmitoylsphingosine	C34H67NO3	1.259506565	
0.00140161	0.1	565.543396	564.535461	28.781	N-Stearoylsphingosine	C36H71NO3	0.008841055	Not significant
0.01055958	0.01055958	643.502319	642.493896	24.8293	Glucosylceramide (d30:1)	C36H69NO8	-0.750580046	10.051282
0.01060669	0.01060669	643.502319	642.494263	24.8048	Glucosylceramide (d30:1)	C36H69NO8	-0.750580046	
0.01151822	0.01151822	744.61	743.605225	23.2883	SM (37:1)	C42H85N2O6P	4.250547266	0.1
0.00962493	0.00962493	744.61	743.605225	23.2883	SM (37:1)	C42H85N2O6P	4.250547266	
0.01041732	0.01041732	647.621643	646.613281	31.8368	Ceramide (d42:2)	C42H81NO3	-0.651615036	10.126068
0.01022029	0.01022029	647.621643	646.613403	31.6768	Ceramide (d42:2)	C42H81NO3	-0.651615036	
0.01930831	0.1	593.574707	592.566956	30.2087	Ceramide (d38:1)	C44H85NO11S	0.318409794	1.723543
0.00507216	0.2	593.574707	592.566833	29.951	Ceramide (d38:1)	C44H85NO11S	0.318409794	
0.00911912	0.00911912	619.59	618.58252	30.2993	Ceramide (d40:2)	C40H77NO3	0.742426443	9.700063
0.00973599	0.00973599	635.621643	634.613159	32.637	Ceramide (d41:1)	C41H81NO3	-0.855855061	8.679758
0.00287013	0.1	563.53	562.519958	28.3715	Ceramide (d36:2)	C36H69NO3	-3.730058737	3.867952
0.01042826	0.01042826	563.527771	562.520203	28.2683	Ceramide (d36:2)	C36H69NO3	-3.730058737	
0.00169133	0.1	565.54	564.536011	28.9343	Ceramide (d36:1)	C36H71NO3	6.986243237	2.109351
0.00941673	0.00941673	835.584351	834.584167	31.0092	3-O-Sulfogalactosylceramide (d38:1)	C44H85NO11S	9.282126922	0.151782
0.01122649	0.2	835.584351	834.584167	31.0092	3-O-Sulfogalactosylceramide (d38:1)	C44H85NO11S	9.282126922	
0.0157015	0.33333333	807.55304	806.553223	29.002	3-O-Sulfogalactosylceramide (36:1)	C42H81NO11S	10.05878202	2.66352
0.01888464	0.2	807.55304	806.553223	29.002	3-O-Sulfogalactosylceramide (36:1)	C42H81NO11S	10.05878202	

Fold UV-DENV/Mock	ID	ID URL
	LMGP06010327	http://www.lipidmaps.org/data/LMSDRecord.php?LMID=LMGP06010193
0.1	LMGP06010327	http://www.lipidmaps.org/data/LMSDRecord.php?LMID=LMGP06010193
	LMGP06010033	http://www.lipidmaps.org/data/LMSDRecord.php?LMID=LMGP06010001
0.116134	LMGP06010033	http://www.lipidmaps.org/data/LMSDRecord.php?LMID=LMGP06010001
Not significant	LMGP06010299	http://www.lipidmaps.org/data/LMSDRecord.php?LMID=LMGP06010105
Not significant	LMGP04020054	http://www.lipidmaps.org/data/LMSDRecord.php?LMID=LMGP04020036
	HMDB11152	http://www.hmdb.ca/metabolites/HMDB11152
0.1	HMDB11152	http://www.hmdb.ca/metabolites/HMDB11152
	LMGP01010008	http://www.lipidmaps.org/data/LMSDRecord.php?LMID=LMGP01010002
0.517361	LMGP01010008	http://www.lipidmaps.org/data/LMSDRecord.php?LMID=LMGP01010002
	HMDB07939	http://www.hmdb.ca/metabolites/HMDB07938
0.51547	HMDB07939	http://www.hmdb.ca/metabolites/HMDB07938
0.1	HMDB08857	http://www.hmdb.ca/metabolites/HMDB08825
0.430265	HMDB07933	http://www.hmdb.ca/metabolites/HMDB07901
	HMDB02396	http://www.hmdb.ca/metabolites/HMDB00220
10.698328	HMDB02396	http://www.hmdb.ca/metabolites/HMDB00220
	HMDB00573	http://www.hmdb.ca/metabolites/HMDB00207
2.526458	HMDB00573	http://www.hmdb.ca/metabolites/HMDB00207
	HMDB00827	http://www.hmdb.ca/metabolites/HMDB00827
0.1	HMDB00827	http://www.hmdb.ca/metabolites/HMDB00827
Not Observed	LMGL01010003	http://www.lipidmaps.org/data/LMSDRecord.php?LMID=LMGL01010003
	HMDB11535	http://www.hmdb.ca/metabolites/HMDB11131
0.54088	HMDB11535	http://www.hmdb.ca/metabolites/HMDB11131
	HMDB11564	http://www.hmdb.ca/metabolites/HMDB11533
0.1	HMDB11564	http://www.hmdb.ca/metabolites/HMDB11533
Not Observed	LMGL02010319	http://www.lipidmaps.org/data/LMSDRecord.php?LMID=LMGL02010319
Not observed	LMGP10010070	http://www.lipidmaps.org/data/LMSDRecord.php?LMID=LMGP10010048
	HMDB04949	http://www.hmdb.ca/metabolites/HMDB00790
0.608542	HMDB04949	http://www.hmdb.ca/metabolites/HMDB00790
0.508831	HMDB04950	http://www.hmdb.ca/metabolites/HMDB00829
	HMDB12320	http://www.hmdb.ca/metabolites/HMDB04969
0.1		
	LMSP03010056	http://www.lipidmaps.org/data/LMSDRecord.php?LMID=LMSP03010055
0.17148	LMSP03010056	http://www.lipidmaps.org/data/LMSDRecord.php?LMID=LMSP03010055
	LMSP02010009	http://www.hmdb.ca/metabolites/HMDB04953
0.1	LMSP02010009	http://www.hmdb.ca/metabolites/HMDB04953
	LMSP02010007	http://www.hmdb.ca/metabolites/HMDB04951
0.513978	LMSP02010007	http://www.hmdb.ca/metabolites/HMDB04951
Not observed	HMDB11775	http://www.lipidmaps.org/data/LMSDRecord.php?LMID=LMSP02010029
Not observed	LMSP02010021	http://www.hmdb.ca/metabolites/HMDB00950
	LMSP02010003	http://www.lipidmaps.org/data/LMSDRecord.php?LMID=LMSP02010003
0.124346	LMSP02010003	http://www.lipidmaps.org/data/LMSDRecord.php?LMID=LMSP02010003
Not significant	LMSP02020015	http://www.lipidmaps.org/data/LMSDRecord.php?LMID=LMSP02010006
	LMSP06020010	http://www.lipidmaps.org/data/LMSDRecord.php?LMID=LMSP06020010
0.51991	LMSP06020010	http://www.lipidmaps.org/data/LMSDRecord.php?LMID=LMSP06020010
	LMSP06020001	http://www.lipidmaps.org/data/LMSDRecord.php?LMID=LMSP06020001
0.45522	LMSP06020001	http://www.lipidmaps.org/data/LMSDRecord.php?LMID=LMSP06020001
