# Supplementary material for: Dengue Virus Infection Perturbs Lipid Homeostasis in Infected Mosquito Cells
Source: PLoS Pathog. 2012 Mar 22;8(3):e1002584. doi: 10.1371/journal.ppat.1002584 (PMC3310792; doi:10.1371/journal.ppat.1002584)
Supplement: Table S3 — Select list of lipid species from the analysis of the 16K membrane fraction (isolated from mosquito cells treated with conditions described below) regulated across conditions (p<0.05). Conditions: DENV (cells infected with DENV in the presence of vehicle only), DENV C75 (cells infected with DENV and treated with the FAS inhibitor C75). A total of 366 features were detected in the mass spectrometry analysis following normalization of DENV and DENV C75 treated samples and 314 of these features were significantly regulated (p<0.05). Identification was successful for 27 lipids (ppm error <10). For each condition the following information is provided: pttest and pwilcox; p-values on minimum observation data (a measurement must be present in two of three replicates to be considered present), Exact mass; mass of each lipid from http://www.lipidmaps.org, M/Z; mass to charge ratio, RT; retention time, Formula, elemental composition of each lipid, PPM Error; difference in experimental mass compared to exact mass, Fold DENV/DENV C75; fold change of average abundance from 3 replicates of each treatment. Identity abbreviations were made for phoshatidylinositol (PI), phosphatidyllglycerol (PG), monoacylglycerol (MG), diacylglycerol (DG), phosphatidic acid (PA). Accession numbers were obtained from the Lipid Maps Gateway (lipidmaps.org) and the Human Metabolome Database (hmdb.ca). (PDF) [file ppat.1002584.s006.pdf]

Supplementary Table 3

p.ttest	p.wilcox	Exact Mass	M/Z	RT	ID (total carbon:double bond)	Formula	PPM Error
2.58896E-05	0.2	836.54	835.532166	21.1125	PI(16:0/18:1(9Z))	C43H81O13P	0.126712411
0.006496139	0.2	834.525818	833.516357	20.3312	PI(16:0/18:2(9Z,12Z))	C43H79O13P	-1.822591905
0.023782329	0.2	808.51	807.501404	20.0265	PI(12:0/20:1(11Z))	C41H77O13P	-0.811369062
0.010137259	0.010137259	856.51	855.501282	19.6338	PI(14:1(9Z)/22:4(7Z,10Z,13Z,16Z))	C45H77O13P	-0.908337322
0.008944193	0.008944193	746.509766	745.50061	20.7662	PG(16:0/18:2(9Z,12Z))	C40H75O10P	-1.62891372
0.016671195	0.2	720.494141	719.485596	20.526	PG(16:0/16:1(9Z))	C38H73O10P	-0.839701485
0.00038947	0.1	537.512085	536.504822	27.7823	N-Palmitoylsphingosine	C34H67NO3	1.259506565
0.001637384	0.1	565.543396	564.536011	28.9343	N-Stearoylsphingosine	C36H71NO3	0.981357052
0.040549168	0.2	643.502319	642.493896	24.8293	Glucosylceramide (d30:1)	C36H69NO8	-0.750580046
0.00136728	0.1	563.527771	562.519958	28.3715	Ceramide (d36:2)	C36H69NO3	0.225366
0.003623002	0.1	593.574707	592.566956	30.2087	Ceramide (d38:1)	C38H75NO3	0.318409794
0.043730735	0.2	647.621643	646.613281	31.8368	Ceramide (d42:2)	C42H81NO3	-0.651615036
0.00974466	0.00974466	535.5	534.489258	27.3163	Cer(d34:2)	C34H65NO3	-5.232492997
0.011095035	0.011095035	619.59	618.58252	30.2993	Cer(d40:2)	C40H77NO3	0.742426443
0.011482263	0.011482263	621.606018	620.597595	31.7678	Ceramide (d40:1)	C40H79NO3	-0.777019504
0.009868462	0.009868462	635.621643	634.613159	32.637	Cer(d41:1)	C41H81NO3	-0.855855061
0.010372965	0.010372965	625.57	624.556091	28.912	DG(36:2)	C39H67D5O5	-9.541697972
0.000909382	0.1	330.277008	329.269653	20.9483	MG(16:0)	C19H38O4	1.771240461
0.001929538	0.1	358.308319	357.300385	32.1102	MG(18:0)	C21H42O4	0.016745355
0.010049983	0.010049983	358.308319	357.300598	30.5262	MG(18:0)	C21H42O4	0.611205457
0.011692959	0.011692959	606.43	605.426941	22.8297	PA(29:0)	C32H63O8P	8.04874429
0.010553801	0.010553801	270.25589	269.248596	21.9505	Heptadecanoic acid	C17H34O2	2.390327182
0.008276665	0.008276665	282.25589	281.248322	8.71102	Oleic acid	C18H34O2	1.317953011
0.01234588	0.01234588	256.240234	255.232941	8.59932	Palmitic acid	C16H32O2	2.524974279
0.011363529	0.011363529	284.271515	283.263763	11.7643	Stearic acid	C18H36O2	0.661339565
0.011852027	0.011852027	785.64	784.632446	30.2037	1,2-dioctadecanoyl-3-O-(6-deoxy-6-amino-alpha-D-glucosyl)-sn-glycerol	C45H87NO9	0.491319179

Fold DENV/DENV C75	ID	ID_url		
4.13392077	LMGP06010001	http://www.lipidmaps.org/data/LMSDRecord.php?LMID=LMGP06010001		
3.44683281	HMDB09784	http://www.hmdb.ca/metabolites/HMDB09784		
4.030520139	LMGP06010027	http://www.lipidmaps.org/data/LMSDRecord.php?LMID=LMGP06010027		
0.1	LMGP06010105	http://www.lipidmaps.org/data/LMSDRecord.php?LMID=LMGP06010105		
0.1	HMDB10575	http://www.hmdb.ca/metabolites/HMDB10575		
5.981845815	HMDB10571	http://www.hmdb.ca/metabolites/HMDB10571		
2.577369226	HMDB00790	http://www.hmdb.ca/metabolites/HMDB00790		
2.242037407	HMDB00829	http://www.hmdb.ca/metabolites/HMDB00829		
2.063881902	HMDB04969	http://www.hmdb.ca/metabolites/HMDB04969		
3.350611523	HMDB04948	http://www.hmdb.ca/metabolites/HMDB04948		
1.679866535	HMDB04951	http://www.hmdb.ca/metabolites/HMDB04951		
1.906851	HMDB04953	http://www.hmdb.ca/metabolites/HMDB04953		
8.936502491	LMSP02010024	http://www.lipidmaps.org/data/LMSDRecord.php?LMID=LMSP02010024		
10.56264764	LMSP02010029	http://www.lipidmaps.org/data/LMSDRecord.php?LMID=LMSP02010029		
9.816951784	HMDB04952	http://www.hmdb.ca/metabolites/HMDB04952		
10.32217057	HMDB00950	http://www.hmdb.ca/metabolites/HMDB00950		
10.34760312	LMGL02010319	http://www.lipidmaps.org/data/LMSDRecord.php?LMID=LMGL02010319		
2.349551054	HMDB11533	http://www.hmdb.ca/metabolites/HMDB11533		
1.661877517	HMDB11131	http://www.hmdb.ca/metabolites/HMDB11131		
9.949724298	HMDB11131	http://www.hmdb.ca/metabolites/HMDB11131		
9.63516104	LMGP10010048	http://www.lipidmaps.org/data/LMSDRecord.php?LMID=LMGP10010048		
10.62041732	HMDB02259	http://www.hmdb.ca/metabolites/HMDB02259		
0.1	HMDB00207	http://www.hmdb.ca/metabolites/HMDB00207		
9.802158788	HMDB00220	http://www.hmdb.ca/metabolites/HMDB00220		
10.89893964	HMDB00827	http://www.hmdb.ca/metabolites/HMDB00827		
0.1	LMGL05010003	http://www.lipidmaps.org/data/LMSDRecord.php?LMID=LMGL05010003		
